# Assessing the severity of cardiovascular disease in 213 088 patients with coronary heart disease: a retrospective cohort study

**DOI:** 10.1136/openhrt-2020-001498

**Published:** 2021-04-20

**Authors:** Salwa S Zghebi, Mamas A Mamas, Darren M Ashcroft, Martin K Rutter, Harm VanMarwijk, Chris Salisbury, Christian D Mallen, Caroline A Chew-Graham, Nadeem Qureshi, Stephen F Weng, Tim Holt, Iain Buchan, Niels Peek, Sally Giles, David Reeves, Evangelos Kontopantelis

**Affiliations:** 1 NIHR School for Primary Care Research, Centre for Primary Care and Health Services Research, Manchester Academic Health Science Centre (MAHSC), The University of Manchester, Manchester, UK; 2 Division of Population Health, Health Services Research and Primary Care, School of Health Sciences, Faculty of Biology, Medicine and Health, Manchester Academic Health Science Centre (MAHSC), The University of Manchester, Manchester, UK; 3 Keele Cardiovascular Research Group, Centre for Prognosis Research, School of Primary, Community and Social Care, Keele University, Stoke-on-Trent, UK; 4 Division of Pharmacy and Optometry, School of Health Sciences, Faculty of Biology, Medicine and Health, Manchester Academic Health Science Centre (MAHSC), The University of Manchester, Manchester, UK; 5 NIHR Greater Manchester Patient Safety Translational Research Centre, The University of Manchester, Manchester, UK; 6 NIHR Manchester Biomedical Research Centre, Manchester Academic Health Science Centre (MAHSC), Manchester, UK; 7 Division of Diabetes, Endocrinology and Gastroenterology, School of Medical Sciences, Faculty of Biology, Medicine and Health, Manchester Academic Health Science Centre (MAHSC), The University of Manchester, Manchester, UK; 8 Diabetes, Endocrinology and Metabolism Centre, Manchester University NHS Foundation Trust, Manchester Academic Health Science Centre (MAHSC), Manchester, UK; 9 Division of Primary Care and Public Health, Brighton and Sussex Medical School, University of Brighton, Brighton, UK; 10 Centre for Academic Primary Care, Population Health Sciences, Bristol Medical School, University of Bristol, Bristol, UK; 11 School of Primary, Community and Social Care, Faculty of Medicine and Health Sciences, Keele University, Staffordshire, UK; 12 Primary Care Stratified Medicine (PRISM) Research Group, Division of Primary Care, School of Medicine, University of Nottingham, Nottingham, UK; 13 Statistical Decision Sciences, Cardiovascular and Metabolism, Janssen Research and Development, High Wycombe, UK; 14 Nuffield Department of Primary Care Health Sciences, University of Oxford, Oxford, UK; 15 Institute of Population Health Sciences, University of Liverpool, Liverpool, UK; 16 Division of Informatics, Imaging and Data Sciences, School of Health Sciences, Faculty of Biology, Medicine and Health, Manchester Academic Health Science Centre (MAHSC), The University of Manchester, Manchester, UK; 17 Centre for Biostatistics, School of Health Sciences, Faculty of Biology, Medicine and Health, Manchester Academic Health Science Centre (MAHSC), The University of Manchester, Manchester, UK

**Keywords:** coronary artery disease, myocardial infarction, electronic health records

## Abstract

**Objective:**

Most current cardiovascular disease (CVD) risk stratification tools are for people without CVD, but very few are for prevalent CVD. In this study, we developed and validated a CVD severity score in people with coronary heart disease (CHD) and evaluated the association between severity and adverse outcomes.

**Methods:**

Primary and secondary care data for 213 088 people with CHD in 398 practices in England between 2007 and 2017 were used. The cohort was randomly divided into training and validation datasets (80%/20%) for the severity model. Using 20 clinical severity indicators (each assigned a weight=1), baseline and longitudinal CVD severity scores were calculated as the sum of indicators. Adjusted Cox and competing-risk regression models were used to estimate risks for all-cause and cause-specific hospitalisation and mortality.

**Results:**

Mean age was 64.5±12.7 years, 46% women, 16% from deprived areas, baseline severity score 1.5±1.2, with higher scores indicating a higher burden of disease. In the training dataset, 138 510 (81%) patients were hospitalised at least once, and 39 944 (23%) patients died. Each 1-unit increase in baseline severity was associated with 41% (95% CI 37% to 45%, area under the receiver operating characteristics (AUROC) curve=0.79) risk for 1 year for all-cause mortality; 59% (95% CI 52% to 67%, AUROC=0.80) for cardiovascular (CV)/diabetes mortality; 27% (95% CI 26% to 28%) for any-cause hospitalisation and 37% (95% CI 36% to 38%) for CV/diabetes hospitalisation. Findings were consistent in the validation dataset.

**Conclusions:**

Higher CVD severity score is associated with higher risks for any-cause and cause-specific hospital admissions and mortality in people with CHD. Our reproducible score based on routinely collected data can help practitioners better prioritise management of people with CHD in primary care.

**Key questions**

**What is already known about this subject?**

- The majority of current cardiovascular disease (CVD) risk stratification tools are for people without CVD with very few tools available for people with prevalent CVD.
- Coronary heart disease (CHD) is the most common type of CVD and a leading cause of death globally. In the UK, CHD is responsible for one death around every 8 min.
- It is estimated that nearly 2.3 million people are living with CHD in the UK.
- The importance of assessing disease severity in people with CHD is well recognised, but validated CVD severity measures derived from routinely collected health records are lacking, as are applications of such measures in primary care settings.

**What does this study add?**

- We developed a new CVD severity score incorporating 20 severity indicators using patients’ anonymised routinely collected electronic health records.
- In people with CHD, a 1-unit higher level of the severity score was linked to up to 59% significantly higher risk of hospital admission or death.

**How might this impact on clinical practice?**

- We demonstrate the utility and validity of a CVD-specific severity measure in people with CHD using routinely collected data.
- Our severity measure has potential applications directly relevant to clinical practice and risk stratification which informs advanced decision making and provides a reproducible algorithm to other conditions managed in primary care.

## Introduction

Cardiovascular disease (CVD) is the leading cause of death globally^1^ and accounts for more than one in four UK deaths.^2^ Coronary heart disease (CHD) is the most common CVD, accounting for nearly 9.5 million deaths worldwide in 2016.^2 3^ Around 15.5 million people had CHD in the USA by 2016, and 2.3 million people in the UK by 2018 at a prevalence of 3%.^2 4^

Most currently available prospective cardiovascular (CV) risk stratification tools are for people without known CVD,^5^ including QRISK and Framingham scores^6 7^ with very few tools available to help assess the disease severity in people with existing CVD. In the context of this paper, we adopt the definition of severity of clinical conditions as the manifestation of the progression of underlying disease processes with implications on healthcare resources utilisation, multimorbidity and mortality.^8 9^

To our knowledge, no established CVD severity scores for primary care patients with CHD exist, and previous cohort studies are sparse,^10^ with the majority of literature based on clinical trials of different sizes or using various sources of data mainly captured in secondary care facilities. Such attempts either focused primarily on existing scores/indices (such as SYNTAX,^11^ Gensini,^12^ the Duke CAD Prognostic Severity Index^13^ and CAD-RADS^14^); the prevalence of multivessel disease; or the degree of coronary stenosis and/or lesions.^13–19^ However, existing scores are not designed for primary care settings and only subserve a small minority of patients. While other scores would need invasive interventions that may not be indicated (or in minority of patients) and therefore resources needed for such information would be limited and not routinely available in primary care settings. CV-specific severity measures derived from routine clinical records of CVD progression are needed and could support practitioners to provide better clinical management as well as help healthcare policy makers and planners in developing services and allocating resources.

Since all of the above approaches rely on data that are not necessarily available in routine primary care health records for all patients, they are not useful for informing decisions at a primary care or public health levels based on identifying patients at risk of adverse outcomes. Currently available routinely collected electronic health records (EHRs) provide a platform for developing disease severity indices that are informative in stratifying CHD populations.

We, therefore, developed a severity score derived from routine EHR in UK primary care to stratify CHD populations in terms of CVD severity as a means of risk stratification. We aimed to: (1) develop and internally validate baseline and longitudinal CV severity scores in individuals with CHD and (2) assess what the score adds to the predictive value of sociodemographic variables for the risks for all-cause and cause-specific hospitalisation and mortality outcomes.

## Methods

### Data source and patient population

In this retrospective cohort study, we used the GOLD database of the Clinical Practice Research Datalink (CPRD). The CPRD is one of the world’s largest EHR databases providing anonymised medical data (including demographics, tests, diagnoses, referrals and prescriptions) and is broadly representative of the UK population.^20 21^ CPRD provides data linkage to additional datasets and disease registries. We used the following linkages: Hospital Episodes Statistics Admitted Patient Care(HES APC), Office for National Statistics (ONS) cause-specific mortality data and index of multiple deprivation (IMD). The IMD used in our study is recorded at the level of the patient’s residential postcode in England and is a score calculated as the weighted sum of 37 individual indicators organised across seven domains of deprivation: access to housing and services, crime, employment, education, income, finance and living environment.^22^ Theemployment and income deprivation domains contribute the most weight to the overall index.

Patients with CHD (defined as patients with ≥1 CHD code listed in online supplemental table S1) aged ≥35 years and registered in linked general practices in England were identified between 1 March 2007 and 31 March 2017. The validity of CVD diagnoses in CPRD data has been acknowledged previously.^23^ For each patient, the index date was defined as the earliest CHD diagnosis date. Patients were followed up until the earliest date of: developing an outcome; leaving the general practice; study end (31 March 2017); or death. By definition of multiple event models (as in Poisson models), developing the outcome of interest was not a censor point for those analyses. The final cohort of eligible patients was randomly split into training (80%) and validation (20%) datasets. The 20% split of the dataset was used to replicate the analyses performed in the training dataset as a validation.

10.1136/openhrt-2020-001498.supp1 Supplementary data

### Severity scores

A scoping review of indicators and markers of disease severity in people with CHD combined by the team’s clinical expert opinion on CVD severity was used to identify clinically relevant CV severity indicators in people with CHD. A total of 20 CV indicators were used: hypertension; hyperlipidaemia; proteinuria/albuminuria; end-stage renal disease; peripheral vascular disease; stable angina; cardiac arrest; atrial fibrillation/supraventricular tachycardia; myocardial infarction/acute coronary syndrome; heart valve disease; endocarditis; myocarditis; cardiomyopathy; pericardial disease; ventricular tachycardia/fibrillation; congestive heart failure; CV procedures; transient ischaemic attack or stroke; diabetes; and pacemaker/defibrillator use. The Read codes for severity indicators recorded in CPRD were identified using the (pcdsearch) Stata command.^24^

Based on the timing of severity indicators, the severity score was calculated as the sum of indicators (each assigned weight=1) recorded at preindex (on/before first CHD diagnosis date, ie, baseline severity) or postindex (after first CHD diagnosis, ie, longitudinal severity) windows (online supplemental table S2). For preindex scores, indicators recorded in three look-back windows were considered: ever before (unlimited look-back window), up to 10 and up to 5 years before index. This aimed to investigate the effects of varying the length of the preindex record on the model fitness in order to identify the optimal look-back window for prediction of future adverse outcomes but simultaneously considering the data quality that improved in recent years. For postindex scores, indicators recorded annually in years 1–10 after index date were considered, each combined with each of the three look-back windows. Postindex scores aimed to assess the trends of CV severity over time and how the risk for adverse outcomes change up to 10 years after CHD diagnosis.

### Covariates

Age at baseline, gender, socioeconomic status (IMD 2015 quintiles 1–5 or unknown) and ethnicity (white, black, Asian, mixed, other or unknown).

### Outcomes

Primary outcome was all-cause mortality. Secondary outcomes were: clustered CV/ diabetes-related mortality; any-cause hospitalisation; clustered CV/diabetes-related hospitalisation; and aggregated any-cause hospitalisation and mortality.

### Data analyses

Cox proportional hazards regression models were fitted to estimate HRs and 95% CIs to assess the relationship between the calculated severity score and outcomes in the training dataset, with the inclusion of sociodemographic covariates. We developed both single event and multiple failure-time events models. The single event models were used to assess the risk for 1-year, 3-year, 5-year and 10-year for each of all-cause mortality and clustered CV/diabetes-related mortality. We experimented with different prediction horizons (1–10 years) to determine how the risks for adverse outcomes change over time after CHD diagnosis. A sensitivity analysis was conducted with the outcome being 1-year all-cause mortality excluding events in first 30 days, as these events may be related to the index event. Multiple failure-time events models were fitted, using the Breslow method to handle tied failures, for the risk of recurrent all-cause hospitalisations. Poisson regression models were used to estimate the unadjusted and adjusted incidence rate ratios (IRRs) and 95% CIs for the association between severity score in a given year and the number of all-cause hospital admissions in the following year annually for 1–10 years after index date. Competing risk analysis was conducted to estimate the subhazard ratio (SHR) and 95% CIs for the risk for 1-year any-cause hospitalisation and 1-year CV/diabetes-related hospitalisation while accounting for deaths as a competing event. Single event Cox models were used to assess the risk for the 1–10 year aggregated any-cause hospitalisation and mortality outcome. Likelihood ratio (LR) tests were fitted to assess the statistical significance of adding each of the developed severity scores (models 2, 3 or 4) to the demographics only model (model 1) in improving the models fit for predicting the outcomes. We also modelled the unlimited severity score divided into four categories: no severity (score=0) as a referent group; low severity (score=1–2); moderate severity (score=3–4); and high severity (score ≥5) to assess the strength of associations between the score and outcomes. Kaplan-Meier survivor function plots for hospitalisation, and mortality outcomes were fitted using severity score categories. All fitted models per outcome are summarised in online supplemental table S3.

The severity scores’ calibration was tested using three methods: Somer’s D^25^; comparing the survival curves for a given risk group^26^; and comparing the observed and predicted survival probabilities in prognostic groups derived by the severity score’s cut points.^27^ Poisson regression, multiple event regression and competing risk models’ goodness of fit was assessed using the Akaike information criterion (AIC), where smaller AIC indicates a better fit of the data than larger AIC.^28^ The predictive value of the single event survival models was assessed using Gönen and Heller’s K concordance statistic (C-statistic), a measure of the area under a receiver operating characteristics (AUROC) curve for censored data.^29^ C-statistic ranges between 0 and 1, where value close to 1 indicates an accurate model with high separation of subjects with different outcomes.^30 31^ Hence, AUROCs are reported for all models except for the three aforementioned models where it was not possible to calculate them. Given the need to use two different postestimation measures (AIC and C-statistic), both were estimated and reported. The proportional hazards assumption was assessed using Schoenfeld residuals. All analyses were replicated in the 20% split of the data as a validation. Data were analysed using Stata software V.15.^32^ The study is reported according to the RECORD checklist.

### Patient and public involvement and engagement (PPIE)

We invited patients with CHD to a PPIE meeting. The participants agreed on the importance and the relevance of the study and suggested the need to raise the awareness about disease severity and to further highlight the fact that it involves several body organs and other conditions. Their perceptions about disease severity and indicators of increased disease severity varied between ‘not thought about disease severity before’ to listing a few indicators they considered relevant, such as declined physical function. The participants shared their views on approaches for disseminating the results via general practices and online social media outlets. We plan to disseminate the study findings widely to patient communities via local heart centres and general practices, and our social media platforms.

## Results

**Figure 1:**
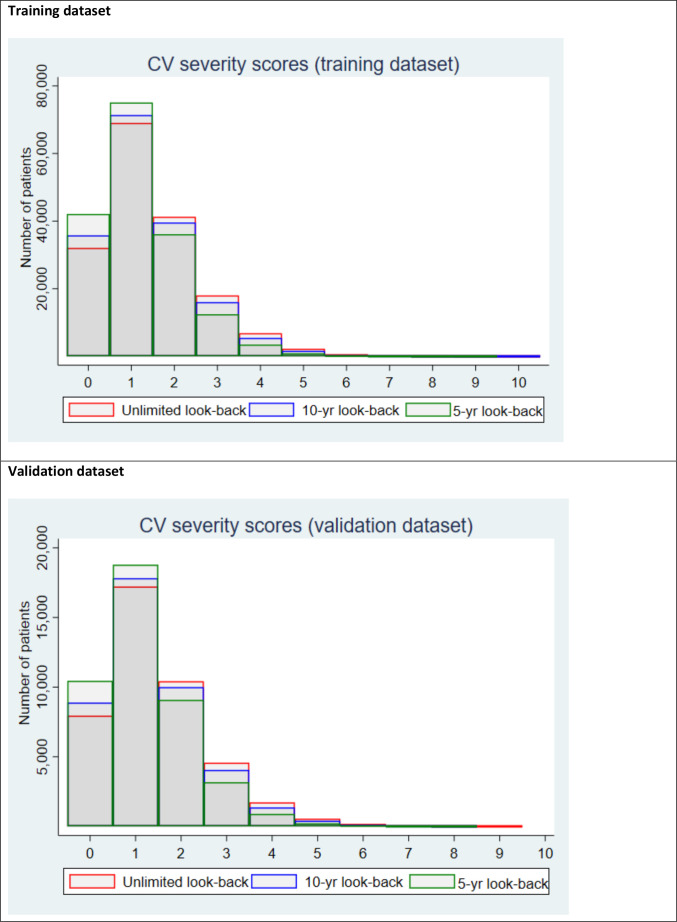
Distribution of baseline cardiovascular (CV) severity scores in the training and validation datasets.

Overall, 213 088 patients with CHD were included (training dataset: n=1 70 395, validation dataset: n=42 693). Mean (±SD) age was 64.5±12.7 years; 46% were women; 89% white; 16% from deprived areas (table 1). The ever before (unlimited) severity score ranged between 0 and 10 (mean±SD: 1.5±1.2), the 10-year before score between 0 and 10 (1.4±1.1) and the 5-year score between 0 and9 (1.2±1.0) (figure 1). The event rates show an increasing event rate with 1-unit increase in baseline unlimited severity score (online supplemental table S4).

**Table 1:** Baseline characteristics of the included patients with coronary heart disease

Characteristic	Full cohort n=213 088	Training dataset n=170 395 (80%)	Validation dataset n=42 693 (20%)
Age (years), mean(±SD)	64.5 (±12.7)	64.5 (±12.7)	64.5 (±12.8)
Gender (female)	98 041 (46.0)	78 444 (46.0)	19 597 (46.0)
Number of general practices	398	398	395
Mean follow-up (years), mean (±SD)	9.4 (±6.0)	9.4 (±6.0)	9.4 (±6.0)
Ethnicity			
White	189 272 (88.8)	151 356 (88.83)	37 916 (88.81)
Black	2017 (0.95)	1626 (0.95)	391 (0.92)
Asian	4933 (2.32)	3940 (2.31)	993 (2.33)
Mixed	596 (0.28)	485 (0.28)	111 (0.26)
Other	1649 (0.77)	1299 (0.76)	350 (0.82)
Unknown	14 621 (6.86)	11 689 (6.86)	2932 (6.87)
Levels of social deprivation (IMD quintiles)			
Q1 (least deprived)	45 719 (21.5)	36 770 (21.6)	8949 (21.0)
Q2	49 251 (23.1)	39 472 (23.2)	9779 (22.9)
Q3	44 543 (20.9)	35 474 (20.8)	9069 (21.2)
Q4	39 032 (18.3)	31 141 (18.3)	7891 (18.5)
Q5 (most deprived)	34 412 (16.2)	27 435 (16.1)	6977 (16.3)
Unknown	131 (0.1)	103 (0.1)	28 (0.1)
Severity indicators at baseline			
Hypertension	109 455 (51.4)	87 422 (51.3)	22 033 (51.6)
Hyperlipidaemia	33 309 (15.6)	26 553 (15.6)	6756 (15.8)
Diabetes	22 763 (10.7)	18 079 (10.6)	4684 (11.0)
Proteinuria/albuminuria	4299 (2.0)	3401 (2.0)	898 (2.1)
End-stage renal disease (ESRD)	623 (0.3)	490 (0.3)	133 (0.3)
Peripheral vascular disease (PVD)	7220 (3.4)	5793 (3.4)	1427 (3.3)
Stable angina	30 838 (14.5)	24 667 (14.5)	6171 (14.5)
Cardiac arrest	1180 (0.6)	919 (0.5)	261 (0.6)
AF/SVT	17 810 (8.4)	14 270 (8.4)	3540 (8.3)
Myocardial infarction/ACS	38 451 (18.0)	30 715 (18.0)	7736 (18.1)
Heart valve disease	3587 (1.7)	2891 (1.7)	696 (1.6)
Endocarditis	292 (0.1)	235 (0.1)	57 (0.1)
Myocarditis	157 (0.1)	118 (0.1)	39 (0.1)
Cardiomyopathy	1105 (0.5)	886 (0.5)	219 (0.5)
Pericardial disease	682 (0.3)	534 (0.3)	148 (0.3)
Ventricular tachycardia/fibrillation	675 (0.3)	519 (0.3)	156 (0.4)
Cardiovascular procedures	10 270 (4.8)	8248 (4.8)	2022 (4.7)
TIA/stroke	18 783 (8.8)	15 053 (8.9)	3730 (8.7)
Pacemaker or defibrillator use	2460 (1.2)	1972 (1.2)	488 (1.1)
Congestive heart failure	10 888 (5.1)	8645 (5.1)	2243 (5.3)

All data are presented as count (%) unless otherwise stated.

ACS, acute coronary syndrome; AF/SVT, atrial fibrillation/supraventricular tachycardia; IMD, Index of multiple deprivation; TIA, transient ischaemic attack.

### All-cause mortality

**Figure 2:**
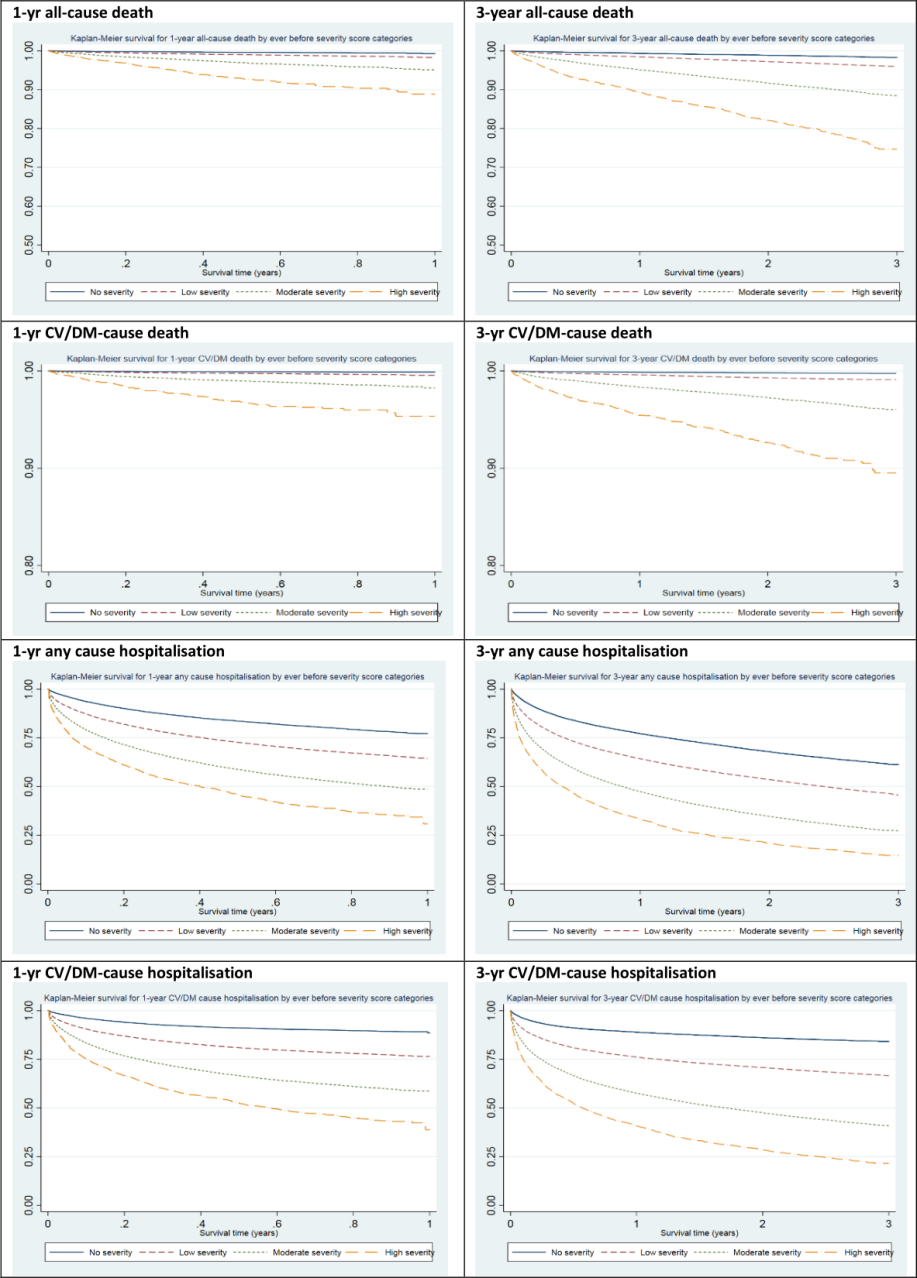
Kaplan-Meier survivor plots for adverse outcomes by CV severity score categories – training dataset. CV, cardiovascular; DM, diabetes mellitus. The survival probability scale (Y-axis) for 1-year and 3-year mortality was curtailed for improved differentiation of survival plots.

Overall, 39 944 deaths occurred in 170 395 patients (23%), of which 1988 (1%) deaths occurred in the first year (of which, 544 events occurred within 30 days) and 24 130 (14%) by the 10th year after index. Higher levels of the severity score was positively associated with increasing risk for all-cause mortality (figure 2A, B). For each one-unit increase of the ever before (unlimited) severity score, the risks for both 1-year and 3-year all-cause mortality increased by 41% (1-year adjusted HR 1.41 (95% CI 1.37 to 1.45, AUROC=0.7912); 3-year HR: 1.41 (95% CI 1.39 to 1.43, AUROC=0.7882), 5 years by 39% (HR: 1.39, 95% CI 1.37 to 1.40, AUROC=0.7872) and 10 years by 35% (HR: 1.35, 95% CI 1.34 to 1.36, AUROC=0.7849). In comparison, the sociodemographics-only model (model 1) had AUROC of 0.7865 for 1-year all-cause mortality, indicating that adding the severity score slightly improved the models predictive value (LR test p<0.0001). The 1–10 year postindex scores showed similar results for risk of all-cause mortality (online supplemental table S5). The sensitivity analysis of excluding deaths in the first 30 days showed similar findings as the primary analysis.

### CV/diabetes-related mortality

Each one unit increase of the unlimited severity score was associated with significantly higher risks at 1, 3, 5 and 10 years: HRs: 1.59 (95% CI 1.52 to 1.67, AUROC=0.8030); 1.61 (95% CI 1.56 to 1.65, AUROC=0.8041); 1.60 (95% CI 1.56 to 1.63, AUROC=0.8024); 1.57 (95% CI 1.55 to 1.60, AUROC=0.8010), respectively (table 2). For 1-year CV/diabetes-related mortality, adding the severity score improved the models predictive value (LR test p<0.0001) in comparison to model 1 (AUROC=0.7962). The 1–10 year postindex scores showed similar trends (online supplemental table S6).

**Table 2:** Survival models for 1, 3, 5 and 10-year CV/diabetes-related mortality using baseline scores—HR (95% CI)—training dataset

	Predictor(s)	1 year	3 years	5 years	10 years
Model 1	Age	1.13 (1.12 to 1.14)	1.13 (1.13 to 1.14)	1.13 (1.13 to 1.13)	1.13 (1.12 to 1.13)
	Gender (F)	0.74 (0.63 to 0.87)	0.70 (0.64 to 0.77)	0.70 (0.65 to 0.75)	0.71 (0.68 to 0.75)
	IMD (vs least deprived)				
	Q5 (most deprived)	1.33 (1.02 to 1.73)	1.47 (1.27 to 1.71)	1.46 (1.30 to 1.64)	1.58 (1.46 to 1.72)
	Ethnicity (vs white)				
	Black	2.80 (1.44 to 5.43)	2.08 (1.35 to 3.21)	1.79 (1.24 to 2.58)	1.45 1.08 to 1.95)
	Asian	1.97 (1.16 to 3.35)	1.80 (1.31 to 2.48)	1.72 (1.33 to 2.22)	1.38 1.13 to 1.70)
	Mixed	–	1.81 (0.75 to 4.35)	1.57 (0.75 to 3.30)	0.81 0.39 to 1.70)
	Other	1.81 (0.81 to 4.05)	1.40 (0.83 to 2.37)	1.30 (0.85 to 2.00)	1.03 (0.73 to 1.45)
	Unknown	1.17 (0.83 to 1.66)	1.22 (1.01 to 1.48)	1.21 (1.04 to 1.41)	1.20 (1.07 to 1.33)
	AUROC	0.7962	0.7961	0.7935	0.7902
	AIC	13 382.17	42 896.17	69 852.12	1 38 058.2
Model 2	Ever before score	1.59 (1.52 to 1.67)	1.61 (1.56 to 1.65)	1.60 (1.56 to 1.63)	1.57 (1.55 to 1.60)
	Age	1.12 (1.11 to 1.13)	1.12 (1.11 to 1.12)	1.12 (1.11 to 1.12)	1.12 (1.11 to 1.12)
	Gender (F)	0.81 (0.69 to 0.95)	0.76 (0.70 to 0.83)	0.76 (0.71 to 0.82)	0.76 (0.72 to 0.80)
	IMD (vs least deprived)				
	Q5 (most deprived)	1.33 (1.02 to 1.73)	1.48 (1.28 to 1.72)	1.47 (1.31 to 1.65)	1.59 (1.46 to 1.72)
	Ethnicity (vs white)				
	Black	2.26 (1.16 to 4.39)	1.67 (1.08 to 2.57)	1.43 (0.99 to 2.07)	1.16 0.87 to 1.56)
	Asian	1.65 (0.97 to 2.80)	1.49 (1.09 to 2.06)	1.43 (1.11 to 1.84)	1.15 0.94 to 1.42)
	Mixed	–	1.81 (0.75 to 4.36)	1.57 (0.75 to 3.29)	0.79 0.37 to 1.65)
	Other	1.73 (0.77 to 3.87)	1.35 (0.80 to 2.29)	1.27 (0.83 to 1.96)	1.05 0.74 to 1.49)
	Unknown	1.49 (1.06 to 2.12)	1.59 (1.31 to 1.93)	1.54 (1.32 to 1.80)	1.48 (1.32 to 1.65)
	AUROC	0.8030	0.8041	0.8024	0.8010
	AIC	13 091.66	41 971.42	68 465.3	AIC=1 35 778.9
Model 3	10-year before score	1.59 (1.51 to 1.67)	1.59 (1.55 to 1.64)	1.58 (1.54 to 1.62)	1.55 (1.53 to 1.58)
	Age	1.12 (1.11 to 1.13)	1.12 (1.12 to 1.13)	1.12 (1.12 to 1.12)	1.12 (1.12 to 1.12)
	Gender (F)	0.81 (0.69 to 0.95)	0.75 (0.69 to 0.83)	0.75 (0.70 to 0.81)	0.75 (0.71 to 0.79)
	IMD (vs least deprived)				
	Q5 (most deprived)	1.32 (1.01 to 1.73)	1.47 (1.27 to 1.71)	1.46 (1.30 to 1.64)	1.57 (1.45 to 1.71)
	Ethnicity (vs white)				
	Black	2.13 (1.19 to 4.50)	1.72 (1.11 to 2.65)	1.48 (1.03 to 2.14)	1.20 1.89 to 1.65)
	Asian	1.74 (1.02 to 2.95)	1.58 (1.15 to 2.17)	1.51 (1.17 to 1.95)	1.20 0.98 to 1.47)
	Mixed	–	1.81 (0.75 to 4.36)	1.57 (0.75 to 3.30)	0.79 0.38 to 1.66)
	Other	1.73 (0.77 to 3.86)	1.38 (0.81 to 2.33)	1.28 (0.83 to 1.97)	1.05 0.74 to 1.48)
	Unknown	1.47 (1.04 to 2.08)	1.55 (1.28 to 1.88)	1.51 (1.29 to 1.75)	1.45 (1.30 to 1.61)
	AUROC	0.8032	0.8039	0.800	0.8000
	AIC	13 133.67	42 115.1	68 675.2	1 36 128.1
Model 4	5-year before score	1.55 (1.46 to 1.65)	1.56 (1.51 to 1.62)	1.56 (1.52 to 1.60)	1.52 (1.49 to 1.55)
	Age	1.12 (1.11 to 1.13)	1.13 (1.12 to 1.13)	1.12 (1.12 to 1.13)	1.12 (1.12 to 1.12)
	Gender (F)	0.79 (0.67 to 0.93)	0.74 (0.68 to 0.81)	0.74 (0.69 to 0.79)	0.74 (0.71 to 0.78)
	IMD (vs least deprived)				
	Q5 (most deprived)	1.32 (1.01 to 1.73)	1.48 (1.27 to 1.71)	1.46 (1.30 to 1.64)	1.58 (1.45 to 1.71)
	Ethnicity (vs White)				
	Black	2.33 (1.20 to 4.54)	1.78 (1.16 to 2.75)	1.57 (1.08 to 2.26)	1.27 (0.95 to 1.71)
	Asian	1.78 (1.05 to 3.03)	1.62 (1.18 to 2.22)	1.55 (1.20 to 2.00)	1.24 1.01 to 1.52)
	Mixed	–	1.83 (0.76 to 4.41)	1.58 (0.75 to 3.31)	0.79 0.38 to 1.66)
	Other	1.75 (0.78 to 3.92)	1.40 (0.82 to 2.36)	1.30 (0.85 to 2.00)	1.06 0.75 to 1.49)
	Unknown	1.41 (1.00 to 2.00)	1.48 (1.22 to 1.80)	1.45 (1.25 to 1.69)	1.40 (1.26 to 1.56)
	AUROC	0.8024	0.8030	0.8010	0.7983
	AIC	13 203.79	42 321.24	68 960.76	1 36 620.3

AIC, Akaike information criterion; AUROC, area under a receiver operating characteristics curve; CV, cardiovascular; DM, diabetes mellitus; IMD, Index of multiple deprivation; SHR, subhazard ratio.

### All-cause hospitalisation

Overall, 138 510 (81% of patients) admissions occurred in 170 395 individuals, of which 43 023 (25%) and 127 358 (75%) occurred within 1 and 10 years after index, respectively. Higher severity showed a greater risk for future all-cause hospitalisation (figure 2E, F). Multiple failure analysis showed an increased risk for recurrent hospitalisation for one-unit increase in score (HR: 1.33, 95% CI 1.29 to 1.37) (online supplemental table S7). For Poisson regression, unadjusted IRRs for the count of next year's hospitalisations ranged between 1.43 (95% CI 1.43 to 1.44) in 1 year after index to 1.37 (95% CI 1.35 to 1.40) in 10 years after index. When also adjusted for covariates, the model fit improved marginally with IRRs ranging between 1.39 (95% CI 1.39 to 1.40) and 1.37 (95% CI 1.34 to 1.40) for the same period (online supplemental table S8). The competing risks analysis showed each one-unit increase of the ever severity score was associated with 27% higher risks for 1-year any-cause hospitalisation (SHR: 1.27 (95% 1.26 to 1.28)) improving the predictive value provided by regression models only including sociodemographic variables – model 1 (LR test p<0.0001) (table 3).

**Table 3:** Competing risk analysis models for 1-year any-cause hospitalisation and 1-year CV/DM-related hospitalisation (competed by all-cause death) using baseline scores – training dataset

	Predictor(s)	1-year any-cause hospitalisation	1-year CV/DM hospitalisation
		SHR (95% CI)	SHR (95% CI)
Model 1	Age	1.02 (1.02 to 1.02)	1.02 (1.02 to 1.02)
	Gender (F)	0.68 (0.67 to 0.70)	0.57 (0.55 to 0.58)
	IMD (vs least deprived)		
	Q5 (most deprived)	1.07 (1.04 to 1.11)	1.05 (1.01 to 1.09)
	Ethnicity (vs white)		
	Black	0.93 (0.84 to 1.03)	0.74 (0.64 to 0.84)
	Asian	1.42 (1.34 to 1.50)	1.53 (1.43 to 1.63)
	Mixed	0.97 (0.81 to 1.17)	1.05 (0.85 to 1.30)
	Other	1.01 (0.91 to 1.13)	1.0 (0.87 to 1.14)
	Unknown	0.22 (0.21 to 0.24)	0.22 (0.20 to 0.24)
	AIC	995 523.4	704 241.7
Model 2	Ever before score	1.27 (1.26 to 1.28)	1.37 (1.36 to 1.38)
	Age	1.01 (1.01 to 1.01)	1.01 (1.01 to 1.01)
	Gender (F)	0.71 (0.70 to 0.73)	0.60 (0.59 to 0.62)
	IMD (vs least deprived)		
	Q5 (most deprived)	1.05 (1.02 to 1.08)	1.02 (0.98 to 1.05)
	Ethnicity (vs white)		
	Black	0.88 (0.79 to 0.97)	0.68 (0.57 to 0.78)
	Asian	1.33 (1.25 to 1.40)	1.39 (1.31 to 1.49)
	Mixed	0.95 (0.79 to 1.14)	1.03 (0.84 to 1.27)
	Other	1.00 (0.90 to 1.12)	0.96 (0.84 to 1.10)
	Unknown	0.24 (0.22 to 0.26)	0.24 (0.22 to 0.27)
	AIC	992 032.8	699 582.5
Model 3	10-year before score	1.28 (1.27 to 1.29)	1.39 (1.37 to 1.40)
	Age	1.01 (1.01 to 1.01)	1.01 (1.01 to 1.01)
	Gender (F)	0.72 (0.70 to 0.73)	0.60 (0.59 to 0.62)
	IMD (vs least deprived)		
	Q5 (most deprived)	1.05 (1.02 to 1.08)	1.01 (0.98 to 1.05)
	Ethnicity (vs white)		
	Black	0.88 (0.80 to 0.97)	0.68 (0.60 to 0.78)
	Asian	1.34 (1.26 to 1.42)	1.40 (1.32 to 1.51)
	Mixed	0.96 (0.80 to 1.15)	1.04 (0.84 to 1.28)
	Other	1.00 (0.90 to 1.12)	0.96 (0.84 to 1.10)
	Unknown	0.24 (0.22 to 0.26)	0.24 (0.22 to 0.27)
	AIC	992 096.3	699 635.1
Model 4	5-year before score	1.30 (1.29 to 1.31)	1.41 (1.40 to 1.43)
	Age	1.01 (1.01 to 1.01)	1.01 (1.01 to 1.01)
	Gender (F)	0.71 (0.70 to 0.73)	0.60 (0.59 to 0.62)
	IMD (vs least deprived)		
	Q5 (most deprived)	1.05 (1.02 to 1.08)	1.02 (0.98 to 1.05)
	Ethnicity (vs white)		
	Black	0.89 (0.80 to 0.98)	0.69 (0.60 to 0.79)
	Asian	1.36 (1.28 to 1.44)	1.44 (1.35 to 1.54)
	Mixed	0.96 (0.80 to 1.16)	1.04 (0.85 to 1.29)
	Other	1.00 (0.90 to 1.12)	0.97 (0.85 to 1.11)
	Unknown	0.24 (0.22 to 0.26)	0.24 (0.22 to 0.27)
	AIC	992 244.2	699 853.5

AIC, Akaike information criterion; CV, cardiovascular; DM, diabetes mellitus; IMD, Index of multiple deprivation; SHR, subhazard ratio.

### CV/diabetes-related hospitalisation

Overall, 30 282 (18% of patients) events occurred within 1 year in 170 395 patients with CHD. For 1-year CV/diabetes-related admissions outcome, each one-unit increase in the ever before severity score was associated with SHR: 1.37 (95% CI 1.36 to 1.38) improving the predictive value provided by model 1 (LR test p<0.0001) (table 3), and it performed better than the any-cause admissions model.

### Aggregated any-cause hospitalisation and mortality

Each one-unit increase in ever before severity score was associated with increased risks by 27% (26%–28%, AUROC=0.6271) at 1 year. Similar trends were observed at 3, 5 and 10 years after index (online supplemental table S9).

A summary of the estimated AIC and AUROC for fitted models is presented in table 4. For models where it was possible to estimate both AIC and AUROC, a summary is plotted in online supplemental figure S1, and there was a trend consistently showing improved model performance predicting cause-specific outcomes over corresponding all-cause outcomes. When categorised, higher severity category levels were associated with increasing risks of hospitalisation and mortality (table 5 and figure 2). Severity score-only models using the training and validation dataset were also fitted and the AUROCs were up to 0.70 as summarised in online supplemental table S10. Models without IMD quintiles are summarised in online supplemental table S11. The performed calibration tests showed good calibration of the severity scores (online supplemental tables S12 and S13, figure 1 vs online supplemental figures S2 and S3). Testing for proportional hazards indicated the assumptions held true (online supplemental figures S4–S8). The validation dataset findings were all similar to those in the training dataset (online supplemental tables S14–S18 and figures S9–S12). The study methods and main findings are outlined in summary online supplemental figures S13 and S14.

**Table 4:** Summary of AIC and AUROCs of fitted Cox and Poisson regression models – training dataset

Predictors/model	1-year all-cause mortality	1-year CV/diabetes-related mortality	Any-cause hospitalisation			1-year CV/diabetes-related hospitalisation*	1-year aggregated any hospitalisation or mortality
			1-year hospitalisation (single event)*	Recurrent event	Poisson† (count in first year)		
**Demographics-only model**							
Model 1	AUROC=0.7865 (AIC=43 050)	AUROC=0.7962 (AIC=13 382)	AIC=995 523	AIC=2.27e+07	AIC=477 990	AIC=704 241	AUROC=0.6055 (AIC=1 008 481)
**Severity score+demographics models**							
Model 2 (model 1+ever before severity score)	AUROC=0.7912 (AIC=42 586.52)	AUROC=0.8030 (AIC=13 091.66)	AIC=992 032.8	AIC=2.26e+07	AIC=456 825	AIC=699 582.5	AUROC=0.6271 (AIC=1 004 865)
Model 3 (model 1+10-year severity score)	AUROC=0.7912 (AIC=42 649.12)	AUROC=0.8032 (AIC=13 133)	AIC=992 096.3	AIC=2.26e+07	AIC=457 753	AIC=699 635	AUROC=0.6270 (AIC=1 004 939)
Model 4 (model 1 + 5-year severity score)	AUROC=0.7910 (AIC=42 732.13)	AUROC=0.8024 (AIC=13 203.79)	AIC=992 244.2	AIC=2.27e+07	AIC=460 109	AIC= 699 853.5	AUROC=0.6265 (AIC=1 005 101)

*Competing risk analysis.

†Adjusted for age, gender and IMD only.

AIC, Akaike information criterion; AUROC, area under a Receiver Operating Characteristics curve; CV, cardiovascular; DM, diabetes mellitus.

**Table 5:** Adjusted 1-year and 3-year HR or SHR (95% CI) for mortality and hospitalisation outcomes by the cardiovascular severity score category

Outcome	Training dataset				Validation dataset			
	No severity	Low severity	Moderate severity	High severity	No severity	Low severity	Moderate severity	High severity
1-year all-cause mortality	Reference	1.49 (1.22 to 1.81)	3.03 (2.46 to 3.72)	5.64 (4.44 to 7.15)	Reference	1.25 (0.87 to 1.80)	2.62 (1.80 to 3.82)	4.76 (3.06 to 7.40)
	**AUROC=0.7899**				**AUROC=0.7881**			
3-year all-cause mortality	Reference	1.40 (1.26 to 1.54)	2.90 (2.61 to 3.22)	5.34 (4.71 to 6.04)	Reference	1.20 (1.00 to 1.45)	2.17 (1.78 to 2.63)	4.63 (3.67 to 5.84)
	**AUROC=0.7863**				**AUROC=0.7857**			
1-year CV/diabetes mortality	Reference	1.58 (1.06 to 2.35)	4.09 (2.73 to 6.11)	9.38 (6.04 to 14.54)	Reference	1.12 (0.58 to 2.18)	3.32 (1.69 to 6.49)	6.53 (3.05 to 13.97)
	**AUROC=0.8003**				**AUROC=0.7951**			
3-year CV/diabetes mortality	Reference	1.95 (1.53 to 2.48)	5.46 (4.28 to 6.96)	11.75 (9.00 to 15.33)	Reference	1.14 (0.78 to 1.68)	2.96 (2.00 to 4.38)	8.53 (5.54 to 13.12)
	**AUROC=0.8038**				**AUROC=0.7953**			
1-year any hospitalisation*	Reference	1.62 (1.57 to 1.67)	2.47 (2.38 to 2.56)	3.47 (3.27 to 3.68)	Reference	1.65 (1.55 to 1.76)	2.51 (2.33 to 2.70)	3.31 (2.94 to 3.73)
	**AIC=992 385.1**				**AIC=2 19 586.1**			
3-year any hospitalisation*	Reference	1.41 (1.38 to 1.44)	2.10 (2.04 to 2.15)	2.85 (2.73 to 2.99)	Reference	1.41 (1.35 to 1.47)	2.05 (1.95 to 2.15)	2.72 (2.48 to 2.97)
	**AIC=1 965 741**				**AIC=4 33 770.1**			
1-year CV/diabetes hospitalisation*	Reference	2.11 (2.02 to 2.20)	3.71 (3.54 to 3.89)	5.51 (5.15 to 5.91)	Reference	2.15 (1.98 to 2.34)	3.73 (3.39 to 4.10)	5.26 (4.59 to 6.04)
	**AIC=6 99 933.1**				**AIC=1 55 656.5**			
3-year CV/diabetes hospitalisation *	Reference	2.03 (1.97 to 2.10)	3.79 (3.66 to 3.92)	5.63 (5.34 to 5.92)	Reference	2.04 (1.92 to 2.17)	3.73 (3.48 to 4.00)	5.45 (4.93 to 6.03)
	**AIC=1 262 175**				**AIC=280 208.9**			

Severity score categories: no severity: score=0 (referent category); low severity: score=1–2; moderate severity: score=3–4; high severity: score ≥5.

All results are adjusted for age, gender, IMD and ethnicity (model 2).

*SHR: subhazard ratio estimated for the competing risk models for risk for hospitalisation.

AIC, Akaike information criterion; AUROC, area under a Receiver Operating Characteristics curve; CV, cardiovascular; IMD, index of multiple deprivation.

## Discussion

### Main findings

In this long-term retrospective cohort study, we present a contemporary and validated scoring system grading CVD severity in people with CHD. Our developed baseline and longitudinal severity scores provide important prognostic information for all-cause and cause-specific hospitalisation and mortality events in people with CHD that had marginal but statistically significant better predictive value in comparison with that provided by models only including sociodemographic variables. Each one-unit increase in disease severity was associated with elevated risks for all-cause mortality by 41%, CV/diabetes mortality by 59% and any cause hospitalisation by 27%.

### Comparison with other studies

A few observational studies have assessed disease severity in people with CHD using routine primary care EHRs, while some studies used data derived from secondary or tertiary care settings to assess severity of CHD for various research questions.^19 33–35^ However, the majority of prior studies assessing the severity of CHD were reporting risk scores based on the anatomical severity and characteristics of CAD, and they are used to assess the prognosis following revascularisation interventions, for example, SYNTAX and Gensini scores,^11 12^ but do not provide information for the majority of patients with CHD not undergoing these interventions.

Some symptom-based tools were reportedly used to categorise disease severity.^36–38^ However, the majority of people with CHD are asymptomatic, which may limit the application of such tools to the wider population of patients with CHD in clinical practice. Other studies classified CHD severity either by the CHD onset type (myocardial infarction, unstable or stable angina categories),^39–41^ or the number of hospitalisation events.^42^

One observational study based on primary care data in Italy, estimated the positive predictive value for automated identification and severity assessment of four chronic conditions, including CHD.^10^ The disease severity in 300 people with CHD was categorised into five levels based on the evidence of presence/absence of heart failure and coronary angioplasty. They reported a good agreement score (Cohen’s kappa=0.69) between the automated algorithm and the general practitioner’s assessment on the CHD severity level.

In our study, the new CV severity score was developed in a larger cohort, and it included heart failure and coronary procedures besides 18 additional severity indicators. Our score can be more applicable to a broader population of patients with CHD than existing scores. Current scores either focus on small and highly selected groups of patients undergoing coronary procedures (eg, SYNTAX)^11^ or define CHD severity in an overall simplistic approach by syndrome that does not take into account the close pathophysiological links between some of the included CV conditions thereby possibly reducing clinical relevance. In addition, we included clinically relevant severity indicators (such as diabetes which contributes to CHD severity^42^), and we evaluated the association between severity score and health outcomes. CV severity indicators may need revising in a few years as newer tests and measures become available in primary care setting. Therefore, future studies can include additional severity indicators, subject to their availability and well recording in primary care data, such as the coronary calcium score, ankle-brachial index test, B-type natriuretic peptide (BNP) or N-terminal pro BNP (NT-pro-BNP) levels and high-sensitivity C reactive protein (hs-CRP) levels. The inclusion of social deprivation data highlights the advantage of used EHRs driven from national healthcare systems, such as the NHS, as patients represent all social levels unlike what would be recorded from private medical systems. While social deprivation levels may not be directly compared with other populations, understanding the underlying domains and the allocation of patients into categories of least deprived versus most deprived may allow for a rough comparison with other populations as appropriate.

### Potential benefits to clinical practice

People with CHD are mainly managed in primary care settings. Our severity measure is based on medical data routinely collected in general practice visits, which indicates its potential usefulness in risk stratification of people with CHD. The score calculation method can be first implemented as a simple table (online supplemental table S2) to enable clinicians estimate patient’s CVD severity at baseline and over time. This can help identify people with CHD at a greater risk for adverse health outcomes, which informs advanced decision making. On a wider context, our algorithm is reproducible for other long-term conditions managed in primary care.

### Study strengths and limitations

The strengths of our study include: first, we analysed a large cohort of patients with CHD to develop and validate the severity scores, derived from a high-quality EHR database. Second, our models were based on baseline and longitudinal severity scores and included important sociodemographic variables, including social deprivation and ethnicity. Third, we compared the added predictive value of the developed score in comparison with that provided by models only including sociodemographic variables in all outcomes. In addition to all-cause mortality, which allows for a broad perspective of the burden of CHD, and hospital admissions, our measured outcomes also included CV and diabetes-related events. Fourth, we used longer term follow-up different from the available 30-day and 6-month risk scores. Finally, we invited people with CHD who provided their feedback on different aspects of the study.

Our study has several limitations. First, there is a risk of misclassifying the identified cases and severity indicators. However, the high validity of CVD diagnoses using CPRD data has been reported previously.^23 43^ Second, other important severity indicators may have been missed since they are not available or routinely recorded in primary care, such as NT-pro-BNP levels or ankle-brachial index. However, using routinely available data allows the creation of a tool that can be applied to primary care and relevant research with EHRs. Third, by the nature of the cohort design, we missed non-survivors (people who died due to the first event). Fourth, as our validation was based on replicated analyses in a separate dataset (internal validation), future study is needed for external validation in an independent database before reporting the complete clinical utility and implications of our score. However, we observed very similar results when we compared our approach to two additional validation analyses based on postestimation from training dataset using CV mortality outcome.^26 44^ Fifth, although the selection of binary weighting system is practical for replication of the score in clinical practice, future studies examining the risks of these indicators considering their different levels of severity, that is, as severity-weighted indicators are required. Finally, generalisability to other healthcare systems and/or other ethnic groups may be limited, but we believe a similar algorithm can be used in those circumstances given that the severity indicators are collected in routine primary care visits.

## Conclusions

While CHD is associated with multiple morbidities and a leading cause of mortality worldwide,^3^ severity measures for CHD based on primary care data are limited and needed. This study provides a contemporary measure of CVD severity derived by routine primary care EHRs for people with CHD, which showed high predictive value of hospitalisation and death outcomes. Our findings indicate that an increase in CVD severity in adult people with CHD was associated with higher risks for all-cause and CV-specific hospital admissions and mortality outcomes. There is underused informative longitudinal, multimorbid structure in routine clinical records and our paper focus on the wider CV spectrum around CHD. Disease-specific severity tools have direct impact on clinical practice, by stratifying care according to disease severity, and can help inform service planning and risk stratification for precision medicine. Future research on external validation of the severity score is needed before reporting its complete clinical utility and implications.

## Data Availability

Clinical code lists are available from clinicalcodes.org. Electronic health records are, by definition, considered sensitive data in the UK by the Data Protection Act and cannot be shared via public deposition because of information governance restriction in place to protect patient confidentiality. Access to data is available only once approval has been obtained through the individual constituent entities controlling access to the data. The primary care data can be requested via application to the Clinical Practice Research Datalink, secondary care data can be requested via application to the Hospital Episode Statistics from the UK Health and Social Care Information Centre, and mortality data are available by application to the UK Office for National Statistics.
