# Supplementary material for: Assessing the severity of cardiovascular disease in 213 088 patients with coronary heart disease: a retrospective cohort study
Source: Open Heart. 2021 Apr 20;8(1):e001498. doi: 10.1136/openhrt-2020-001498 (PMC8061853; doi:10.1136/openhrt-2020-001498)

Supplementary data to '*Assessing the severity of CVD in 213,088 patients with CHD'- Zghebi et al. 2020*

SUPPLEMENTAL MATERIAL

Zghebi SS, *et al. Open Heart* 2021; 8:e001498. doi: 10.1136/openhrt-2020-001498

Supplementary data to '*Assessing the severity of CVD in 213,088 patients with CHD'- Zghebi et al. 2020*

Table S1 inclusion codes for CHD

	Medical code	Read code	Description	Medical code	Read code	Description	Medical code	Read code	Description
1.	240	G3...00	Ischaemic heart disease	29902	G330z00	Angina decubitus NOS	102943	8Hkl.00	Referral to cardiac rehabilitation service by secondary care
2.	241	G30..00	Acute myocardial infarction	30171	G5...00	Other forms of heart disease	103046	G210z00	Malignant hypertensive heart disease NOS
3.	1021	5543.00	Coronary arteriograph.abnormal	30330	G309.00	Acute Q-wave infarct	103655	187..00	Frequency of angina
4.	1204	G30..14	Heart attack	30421	G30..13	Cardiac rupture following myocardial infarction (MI)	103932	8CMP.00	Coronary heart disease care plan
5.	1344	G340.12	Coronary artery disease	30963	1J61.00	Suspected ischaemic heart disease	104675	8F97.00	Cardiac rehabilitation programme completed
6.	1414	G33z300	Angina on effort	31464	G21z.00	Hypertensive heart disease NOS	105216	14AW.00	H/O acute coronary syndrome
7.	1430	G33..00	Angina pectoris	32272	G38..00	Postoperative myocardial infarction	105250	G341111	Mural cardiac aneurysm
8.	1431	G311.13	Unstable angina	32450	G33z400	Ischaemic chest pain	105479	G39..00	Coronary microvascular disease
9.	1490	G5z..00	Heart disease NOS	32526	14AA.00	H/O: heart disease NOS	105615	G01yz00	Other acute rheumatic heart disease NOS
10.	1537	662..11	Heart disease monitoring	32854	G30B.00	Acute posterolateral myocardial infarction	105938	G211z00	Benign hypertensive heart disease NOS
11.	1655	G340.11	Triple vessel disease of the heart	34207	9Ob4.00	Coronary heart disease monitoring 2nd letter	106812	G383.00	Postoperative transmural myocardial infarction unspec site
12.	1676	G3z..00	Ischaemic heart disease NOS	34328	G311300	Refractory angina	107574	8T04.00	Referral to Angina Plan self-management programme
13.	1677	G30..15	MI - acute myocardial infarction	34329	9Ob5.00	Coronary heart disease monitoring 3rd letter	107967	661M000	Angina self-management plan agreed
14.	1678	G308.00	Inferior myocardial infarction NOS	34633	G34y.00	Other specified chronic ischaemic heart disease	108056	8IEY.00	Referral to Angina Plan self-management programme declined
15.	1792	G3...13	IHD - Ischaemic heart disease	34803	G30y.00	Other acute myocardial infarction	109035	Gyu3500	[X]Subsequent myocardial infarction of other sites
16.	1811	G5yz.00	Other heart disease NOS	34952	32B..00	ECG: Q wave	110535	8F98.00	Cardiac rehabilitation programme offered
17.	2155	G341000	Ventricular cardiac aneurysm	35119	G501.00	Post infarction pericarditis	110634	Gyu4000	[X]Other specified pulmonary heart diseases
18.	2491	G30..12	Coronary thrombosis	35277	9Ob1.00	Refuses coronary heart disease monitoring	25583	G574011	Cardiac arrest-ventricular fibrillation
19.	3468	662..00	Cardiac disease monitoring	35287	322Z.00	ECG: myocardial ischaemia NOS	25814	9Ob3.00	Coronary heart disease monitoring 1st letter

Zghebi SS, *et al. Open Heart* 2021; 8:e001498. doi: 10.1136/openhrt-2020-001498

Supplementary data to '*Assessing the severity of CVD in 213,088 patients with CHD*'- *Zghebi et al. 2020*

	Medical code	Read code	Description	Medical code	Read code	Description	Medical code	Read code	Description
20.	3704	G307.00	Acute subendocardial infarction	35373	9Ob0.00	Attends coronary heart disease monitoring	25842	G33z.00	Angina pectoris NOS
21.	3999	G340000	Single coronary vessel disease	35382	5533.00	Angiocardiography abnormal	26863	G33z600	New onset angina
22.	4017	G32..00	Old myocardial infarction	35674	14A3.00	H/O: myocardial infarct <60	26972	3234.00	ECG:posterior/inferior infarct
23.	4656	G311.11	Crescendo angina	35713	G34yz00	Other specified chronic ischaemic heart disease NOS	26973	3222.00	ECG:shows myocardial ischaemia
24.	5221	44H3.00	Cardiac enzymes abnormal	36193	G5y..00	Other specified heart disease	26975	3233.00	ECG: antero-septal infarct.
25.	5254	G340100	Double coronary vessel disease	36423	G36..00	Certain current complication follow acute myocardial infarct	27484	G341.11	Cardiac aneurysm
26.	5387	G301.00	Other specified anterior myocardial infarction	36523	G311.00	Preinfarction syndrome	27951	G31..00	Other acute and subacute ischaemic heart disease
27.	5413	G340.00	Coronary atherosclerosis	36609	G342.00	Atherosclerotic cardiovascular disease	27977	G31yz00	Other acute and subacute ischaemic heart disease NOS
28.	6331	G341.00	Aneurysm of heart	36854	G332.00	Coronary artery spasm	28138	G34..00	Other chronic ischaemic heart disease
29.	6336	14A5.00	H/O: angina pectoris	37657	G362.00	Ventric septal defect/curr comp fol acut myocardal infarctn	28554	G33zz00	Angina pectoris NOS
30.	7320	G343.00	Ischaemic cardiomyopathy	37990	8F90.00	Cardiac rehabilitation - phase 1	28736	G30y000	Acute atrial infarction
31.	7347	G311100	Unstable angina	37991	8F91.00	Cardiac rehabilitation - phase 2	29300	662K300	Angina control - worsening
32.	7696	G33z200	Syncope anginosa	38609	G351.00	Subsequent myocardial infarction of inferior wall	29421	G344.00	Silent myocardial ischaemia
33.	7783	323..00	ECG: myocardial infarction	39449	G312.00	Coronary thrombosis not resulting in myocardial infarction	29553	G366.00	Thrombosis atrium,auric append&vent/curr comp foll acute MI
34.	8246	322..00	ECG: myocardial ischaemia	39500	9Ob8.00	Coronary heart disease monitoring check done	29643	G303.00	Acute inferoposterior infarction
35.	8516	8F9..00	Cardiac rehabilitation	39546	Gyu3000	[X]Other forms of angina pectoris	29758	G30X.00	Acute transmural myocardial infarction of unspecif site
36.	8568	G37..00	Cardiac syndrome X	39655	G311.12	Impending infarction	37908	9Ob6.00	Coronary heart disease monitoring verbal invitation
37.	8935	G302.00	Acute inferolateral infarction	39693	G31y200	Subendocardial ischaemia	102914	8IE3.00	Referral to cardiac rehabilitation programme declined
38.	9276	G31y000	Acute coronary insufficiency	39904	3232.00	ECG: old myocardial infarction	66388	G33z000	Status anginosus
39.	9413	G31y.00	Other acute and subacute ischaemic heart disease	40399	14A4.00	H/O: myocardial infarct >60	67087	G341100	Other cardiac wall aneurysm
40.	9507	G307000	Acute non-Q wave infarction	40429	G301000	Acute anteroapical infarction	68357	G31y100	Microinfarction of heart
41.	9555	G33z500	Post infarct angina	40624	ZL62200	Referral to cardiac rehabilitation nurse	68401	Gyu3200	[X]Other forms of acute ischaemic heart disease
42.	10109	G....13	Heart diseases	41032	8F92.00	Cardiac rehabilitation - phase 3	68748	G38z.00	Postoperative myocardial infarction, unspecified

Supplementary data to ‘*Assessing the severity of CVD in 213,088 patients with CHD’- Zghebi et al. 2020*

	Medical code	Read code	Description	Medical code	Read code	Description	Medical code	Read code	Description
43.	10127	8H7v.00	Referral to cardiac rehabilitation nurse	41179	G5yyz00	Other ill-defined heart disease NOS	68849	G01z.00	Acute rheumatic heart disease NOS
44.	10260	6A4..00	Coronary heart disease review	41221	G30y200	Acute septal infarction	68979	Gyu5.00	[X]Other forms of heart disease
45.	10562	G307100	Acute non-ST segment elevation myocardial infarction	41677	G341z00	Aneurysm of heart NOS	69474	G365.00	Rupture papillary muscle/curr comp fol acute myocard infarct
46.	11048	G331.11	Variant angina pectoris	41835	G384.00	Postoperative subendocardial myocardial infarction	69776	SP00300	Mechanical complication of coronary bypass
47.	11648	8B3k.00	Coronary heart disease medication review	42104	32E4.00	ECG: S-T depression	70160	9Ob9.00	Coronary heart disease monitoring telephone invite
48.	11983	G311500	Acute coronary syndrome	45476	14AL.00	H/O: Treatment for ischaemic heart disease	71046	G41yz00	Other chronic pulmonary heart disease NOS
49.	12139	G300.00	Acute anterolateral infarction	45809	G350.00	Subsequent myocardial infarction of anterior wall	72562	G353.00	Subsequent myocardial infarction of other sites
50.	12229	G30X000	Acute ST segment elevation myocardial infarction	45960	8B27.00	Antianginal therapy	91774	G341300	Acquired atrioventricular fistula of heart
51.	12804	G33z700	Stable angina	46017	G30yz00	Other acute myocardial infarction NOS	95550	8H2V.00	Admit ischaemic heart disease emergency
52.	12986	G331.00	Prinzmetal's angina	13250	G....12	Cardiac diseases	97001	44p2.00	Cardiac troponin positive
53.	13185	662K.00	Angina control	46166	G35X.00	Subsequent myocardial infarction of unspecified site	99991	Gyu3600	[X]Subsequent myocardial infarction of unspecified site
54.	13187	662N.00	CHD monitoring	46227	32B2.00	ECG: Q wave abnormal	100139	14AT.00	History of myocardial infarction
55.	46112	G380.00	Postoperative transmural myocardial infarction anterior wall	46276	G381.00	Postoperative transmural myocardial infarction inferior wall	101164	8LF..00	Coronary angiography planned
56.	13566	G30..11	Attack - heart	46565	8F93.00	Cardiac rehabilitation - phase 4	101373	8L41.00	Coronary angioplasty planned
57.	13571	G30..16	Thrombosis - coronary	47637	Gyu3300	[X]Other forms of chronic ischaemic heart disease	102447	8Hkk.00	Referral to cardiac rehabilitation programme
58.	14658	G30z.00	Acute myocardial infarction NOS	47798	9Ob2.00	Coronary heart disease monitoring default	19067	ZL22200	Under care of cardiac rehabilitation nurse
59.	14782	662K200	Angina control - improving	48981	66f1.00	Cardiovascular disease interim monitoring	19185	66f..00	Cardiovascular disease monitoring
60.	14897	G301z00	Anterior myocardial infarction NOS	50372	14AH.00	H/O: Myocardial infarction in last year	19250	8I3a.00	Cardiac rehabilitation declined
61.	14898	G305.00	Lateral myocardial infarction NOS	51043	ZRBN.00	Duke's coronary artery disease score	19542	662K000	Angina control - good
62.	15349	662Kz00	Angina control NOS	52517	Gyu3.00	[X]Ischaemic heart diseases	19655	G311.14	Angina at rest
63.	15373	662K100	Angina control - poor	52705	3236.00	ECG: lateral infarction	19744	8I37.00	Coronary heart disease monitoring refused
64.	15661	G310.11	Dressler's syndrome	54251	G311z00	Preinfarction syndrome NOS	19827	3213111	Positive exercise ECG test

Zghebi SS, *et al. Open Heart* 2021; 8:e001498. doi: 10.1136/openhrt-2020-001498

Supplementary data to '*Assessing the severity of CVD in 213,088 patients with CHD'- Zghebi et al. 2020*

	Medical code	Read code	Description	Medical code	Read code	Description	Medical code	Read code	Description
65.	15754	G34z.00	Other chronic ischaemic heart disease NOS	54535	G33z100	Stenocardia	20001	G1z..00	Chronic rheumatic heart disease NOS
66.	15782	G41z.00	Chronic pulmonary heart disease NOS	55137	G311011	MI - myocardial infarction aborted	20095	G330.00	Angina decubitus
67.	16173	G21zz00	Hypertensive heart disease NOS	55401	3235.00	ECG: subendocardial infarct	20416	G3...12	Atherosclerotic heart disease
68.	16408	G32..11	Healed myocardial infarction	57062	14AJ.00	H/O: Angina in last year	21844	G31y300	Transient myocardial ischaemia
69.	16657	ZV7B011	[V]Screening for ischaemic heart disease (IHD)	59032	323Z.00	ECG: myocardial infarct NOS	22383	G3y..00	Other specified ischaemic heart disease
70.	17133	G30A.00	Mural thrombosis	59189	G363.00	Ruptur cardiac wall w'out haemopericard/cur comp fol ac MI	23078	G34y100	Chronic myocardial ischaemia
71.	17307	G311200	Angina at rest	59193	G341200	Aneurysm of coronary vessels	23098	ZV57900	[V]Cardiac rehabilitation
72.	17464	G32..12	Personal history of myocardial infarction	59854	G1yzz00	Other rheumatic heart disease NOS	23579	G310.00	Postmyocardial infarction syndrome
73.	17681	662Z.00	Cardiac disease monitoring NOS	59940	G364.00	Ruptur chordae tendinae/curr comp fol acute myocard infarct	23708	G361.00	Atrial septal defect/curr comp folow acut myocardal infarct
74.	17689	G30..17	Silent myocardial infarction	60664	44H3000	Cardiac enzymes abnormal - first set	23892	G304.00	Posterior myocardial infarction NOS
75.	17872	G301100	Acute anteroseptal infarction	61166	G21z000	Hypertensive heart disease NOS without CCF	24126	G360.00	Haemopericardium/current comp folow acut myocard infarct
76.	18118	G311400	Worsening angina	61670	889A.00	Diab mellit insulin-glucose infus acute myocardial infarct	24540	G34y000	Chronic coronary insufficiency
77.	18125	G330000	Nocturnal angina	62270	32B3.00	ECG: Q wave pathological	24783	G3...11	Arteriosclerotic heart disease
78.	18134	182A.00	Chest pain on exertion	62626	G30y100	Acute papillary muscle infarction	19044	ZLA2200	Seen by cardiac rehabilitation nurse
79.	18135	6A2..00	Coronary heart disease annual review	62718	G21z100	Hypertensive heart disease NOS with CCF	66285	32BZ.00	ECG: Q wave NOS
80.	18150	9Ob..00	Coronary heart disease monitoring administration	63467	G306.00	True posterior myocardial infarction	18889	G34z000	Asymptomatic coronary heart disease
81.	18218	Z677.00	Cardiac rehabilitation class	63538	32B1.00	ECG: Q wave normal	65533	G40z.00	Acute pulmonary heart disease NOS
82.	18842	G35..00	Subsequent myocardial infarction						

Supplementary data to '*Assessing the severity of CVD in 213,088 patients with CHD*'- *Zghebi et al. 2020*

Table S2 Simple illustration of pre-index and post-index CVD score calculation in people with CHD

**Patient ID**	**CHD diagnosis**	**Diagnosis of severity indicator**	**Pre-index severity indicator eligibility at**			**Post-index severity indicator eligibility at post-index (e.g. 3-year post-index) +**		
			5-yr look-back window	10- yr look-back window	Unlimited look-back window	5-yr look-back window[#]	10- yr look-back window[^]	Unlimited look-back window[&]
**0001**	11/07/2007	PVD on 13/10/1992	N	N	**Y**	N	N	**Y**
		TIA on 01/03/1994	N	N	**Y**	N	N	**Y**
		Stroke on 22/08/2001	N	**Y**	**Y**	N	**Y**	**Y**
		DM on 06/12/2005	**Y**	**Y**	**Y**	**Y**	**Y**	**Y**
		MI on 18/03/2010	-	-	-	**Y**	**Y**	**Y**
	**Score / window**		**1**	**2**	**4**	**2**	**3**	**5**
**0002**	19/08/2011	CABG on 28/01/2014	-	-	-	**Y**	**Y**	**Y**
	**Score / window**		**0**	**0**	**0**	**1**	**1**	**1**
**0003**	03/02/2015	HT on 27/05/2002	N	N	**Y**	N	N	**Y**
		ESRD on 14/12/2016	-	-	-	**Y**	**Y**	**Y**
	**Score / window**		**0**	**0**	**1**	**1**	**1**	**2**

These data are based on fictional patient IDs and presented for illustrative purpose only.
[#] this covers 3 years after index and 2 years before index i.e. total of 5 years window.
[^] this covers 3 years after index and 7 years before index i.e. total of 10 years window.
[&] this covers 3 years after index and unlimited window before index.

CABG: coronary artery bypass graft; **DM**: diabetes mellitus; **ESRD**: end stage renal disease; **HT**: hypertension; **MI**: myocardial infarction; **PVD**: peripheral vascular disease; **TIA**: transient ischaemic attack.

So, we computed a total of 33 pre-index and post-index severity scores using different study windows for each patient:

- Three pre-index severity scores for computing overall severity using the three look-back windows:
  - Unlimited look-back window
  - 10-year look-back window
  - 5-year look-back window

- 30 post-index severity scores, based on combining each of windows 1-3 above with post-index windows of length of 1-10 years. For example, for the 1-year post-index window:
  - 1-year post-index window combined with unlimited look-back window
  - 1-year post-index window combined with 10-year look-back window
  - 1-year post-index window combined with 5-year look-back window
  - And so on for the 2-10 years post-index windows.

Supplementary data to '*Assessing the severity of CVD in 213,088 patients with CHD'- Zghebi et al. 2020*

Table S3 A total of 212 Cox and Poisson models were fitted for primary and secondary outcomes.

		All-cause death	Clustered CV- or Diabetes-related death	Any cause hospitalisation	Clustered CV/Diabetes-hospitalisation	Aggregated any hospitalisation or mortality	Recurrent hospitalisation (multiple event)
**Baseline CVD scores (Cox models)**							
	Model 1 (Age, gender, ethnicity, and IMD)	√ √ √ √	√ √ √ √	√ √ √ √	√ √ √ √	√ √ √ √	√
**Model 2**	Model 1 + Unlimited score	√ √ √ √	√ √ √ √	√ √ √ √	√ √ √ √	√ √ √ √	√
	Model 1 + 10-year before score	√ √ √ √	√ √ √ √	√ √ √ √	√ √ √ √	√ √ √ √	√
	Model 1 + 5-year before score	√ √ √ √	√ √ √ √	√ √ √ √	√ √ √ √	√ √ √ √	√
**Baseline CVD score categories (Cox models)**		√ √	√ √	√ √	√ √	-	-
**Moving CVD scores (Cox models)**							
	Model 1 (Age, gender, ethnicity, and IMD) between index+1year to index+10 years	√ √ √ √ √ √ √ √ √ √ √	√ √ √ √ √ √ √ √ √ √ √	-	-	-	-
**Model 2**	Model 1 + Unlimited scores between index+1year to index+10 years	√ √ √ √ √ √ √ √ √ √ √	√ √ √ √ √ √ √ √ √ √ √	-	-	-	-
	Model 1 + 10-year before scores between index+1year to index+10 years	√ √ √ √ √ √ √ √ √ √ √	√ √ √ √ √ √ √ √ √ √ √	-	-	-	-
	Model 1 + 5-year before scores between index+1year to index+10 years	√ √ √ √ √ √ √ √ √ √ √	√ √ √ √ √ √ √ √ √ √ √	-	-	-	-
**Moving CVD scores (Poisson models)**							
	Model 1 (Age, gender, ethnicity, and IMD) at index+1year to index+10 years	-	-	√ √ √ √ √ √ √ √ √ √ √	-	-	-
**Model 2**	Model 1 + Unlimited scores at index+1year to index+10 years	-	-	√ √ √ √ √ √ √ √ √ √ √	-	-	-
	Model 1 + 10-year before scores at index+1year to index+10 years	-	-	√ √ √ √ √ √ √ √ √ √ √	-	-	-
	Model 1 + 5-year before scores at index+1year to index+10 years	-	-	√ √ √ √ √ √ √ √ √ √ √	-	-	-

Zghebi SS, *et al. Open Heart* 2021; 8:e001498. doi: 10.1136/openhrt-2020-001498

Supplementary data to '*Assessing the severity of CVD in 213,088 patients with CHD*'- *Zghebi et al. 2020*

Table S4 Absolute event rates (%) per CV severity score strata – Training and validation datasets

Unlimited score	1-year all-cause mortality	10-year all-cause mortality	1-year CV_DM mortality	10-year CV_DM mortality	1-year any-cause hospitalisation	10-year any-cause hospitalisation	1-year CV_DM hospitalisation	10-year CV_DM hospitalisation
**Training dataset**								
**0**	0.3	6.2	0.09	1.3	14.9	62.8	7.9	27.0
**1**	0.7	10.9	0.17	2.5	22.3	73.0	14.7	43.6
**2**	1.3	16.7	0.47	4.7	29.1	79.5	21.4	59.5
**3**	2.3	23.3	0.89	7.7	35.7	84.6	28.3	71.2
**4**	3.9	32.3	1.46	11.4	41.8	87.0	34.9	78.3
**5**	5.7	39.0	2.17	14.9	47.3	89.5	40.5	84.7
**6**	7.0	45.2	4.10	19.0	50.9	87.9	44.6	82.6
**7**	9.6	50.0	5.15	23.7	54.5	92.1	51.7	94.8
**8**	14.3	62.9	10.53	31.6	71.4	94.3	62.9	89.5
**9**	11.1	88.9	0.00	33.3	77.8	88.9	55.6	83.3
**10**	33.3	100.0	0.00	0.0	66.7	66.7	66.7	100.0
**Overall**	**1.2**	**14.2**	**0.36**	**3.7**	**25.2**	**74.7**	**17.8**	**48.0**
**Validation dataset**								
**0**	0.4	6.1	0.13	1.3	14.8	63.3	7.8	25.4
**1**	0.7	10.6	0.20	2.2	22.2	72.4	14.5	42.0
**2**	1.3	16.8	0.35	3.9	29.4	79.6	22.0	57.3
**3**	2.4	24.2	0.83	7.7	36.1	85.1	28.7	70.7
**4**	4.7	31.1	2.03	10.5	41.8	86.2	34.4	77.1
**5**	5.3	38.2	2.36	13.3	44.9	89.8	38.4	80.5
**6**	9.7	57.8	3.24	23.2	48.6	85.9	43.2	82.7
**7**	8.7	41.3	4.35	26.1	65.2	95.7	63.0	95.7
**8**	16.7	66.7	8.33	16.7	58.3	91.7	41.7	83.3
**9**	0.0	66.7	0.00	66.7	100.0	100.0	66.7	100.0
**Overall**	**1.2**	**14.2**	**0.41**	**3.6**	**25.3**	**74.7**	**17.9**	**47.9**

Zghebi SS, *et al. Open Heart* 2021; 8:e001498. doi: 10.1136/openhrt-2020-001498

Supplementary data to '*Assessing the severity of CVD in 213,088 patients with CHD*'- *Zghebi et al. 2020*

Table S5 Cox regression models using moving CV severity scores for all-cause mortality HR (95% CI) – for 2-9 yrs windows models only adjusted HRs for severity scores are presented for simplicity – training dataset

Al-cause death		Index+1 year	Index+2 years	Index+3 years	Index+4 years	Index+5 years	Index+6 years	Index+7 years	Index+8 years	Index+9 years	Index+10 years
**Age Gender IMD, ethnicity Only**	**Model 1**	**AUROC**=0.7744<br>AIC=848,222.2	**AUROC**= 0.7722<br>AIC= 803,473.9	**AUROC**=0.7697<br>AIC= 745,513.2	**AUROC**=0.7669<br>AIC=691,389.6	**AUROC**=0.7645<br>AIC= 638,300.5	**AUROC**=0.7622<br>AIC= 581,226.6	**AUROC**=0.7598<br>AIC=526,475.7	**AUROC**= 0.7572<br>AIC=470,098.2	**AUROC**= 0.7552<br>AIC=415,919.8	**AUROC**=0.7531<br>AIC=362,958.9
**+Ever before score**	**Model 2**	• Score<br>1.28 (1.27; 1.29)<br>• Age<br>1.11 (1.11; 1.11)<br>• Gender (F) 0.73 (0.72; 0.75)<br>• IMD (Q5)<br>1.58 (1.53; 1.63)<br>• Race (Asian)<br>0.90 (0.81; 0.98)<br>**AUROC**= 0.7822<br>AIC= 844,464.5	1.25 (1.24; 1.26)<br><br>**AUROC**=0.7802<br>AIC= 800,104.8	1.24 (1.23; 1.25)<br><br>**AUROC**=0.7776<br>AIC= 742,514.6	1.23 (1.22; 1.24)<br><br>**AUROC**=0.7747<br>AIC= 688,677	1.22 (1.21; 1.23)<br><br>**AUROC**=0.7722<br>AIC=635,824.1	1.21 (1.21; 1.22)<br><br>**AUROC**=0.7701<br>AIC= 578,906.6	1.21 (1.20; 1.22)<br><br>**AUROC**=0.7676<br>AIC=524,377.8	1.21 (1.20; 1.22)<br><br>**AUROC**=0.7654<br>AIC= 468,110.3	1.21 (1.20; 1.22)<br><br>**AUROC**=0.7640<br>AIC=414,083.5	• Score<br>1.21 (1.20; 1.22)<br>• Age<br>1.10 (1.10; 1.11)<br>• Gender (F) 0.76 (0.73; 0.78)<br>• IMD (Q5)<br>1.51 (1.44; 1.59)<br>• Race (Asian)<br>0.74 (0.64; 0.87)<br>**AUROC**=0.7620<br>AIC= 361,310.1
**+10 yrs before score**	**Model 3**	• Score<br>1.27 (1.25; 1.27)<br>• Age<br>1.11 (1.11; 1.11)<br>• Gender (F) 0.73 (0.71; 0.74)<br>• IMD (Q5)<br>1.57 (1.52; 1.63)<br>• Race (Asian)<br>0.91 (0.83; 1.00)<br>**AUROC**=0.7811<br>AIC= 845,152.5	1.23 (1.22; 1.24)<br><br>**AUROC**=0.7789<br>AIC= 800,781.2	1.22 (1.21 1.23)<br><br>**AUROC**=0.7762<br>AIC= 743,106.9	1.21 (1.20; 1.22)<br><br>**AUROC**=0.7733<br>AIC= 689,221.3	1.20 (1.19; 1.21)<br><br>**AUROC**=0.7707<br>AIC=636,353	1.20 (1.19; 1.20)<br><br>**AUROC**=0.7686<br>AIC=579,404.8	1.19 (1.18; 1.20)<br><br>**AUROC**=0.7660<br>AIC=524,842.3	1.19 (1.18; 1.20)<br><br>**AUROC**=0.7637<br>AIC= 468,527.3	1.19 (1.18; 1.20)<br><br>**AUROC**=0.7621<br>AIC=414,480.9	• Score<br>1.19 (1.18; 1.20)<br>• Age<br>1.10 (1.10; 1.11)<br>• Gender (F) 0.75 (0.72; 0.77)<br>• IMD (Q5)<br>1.52 (1.44; 1.59)<br>• Race (Asian)<br>0.76 (0.65; 0.89)<br>**AUROC**=0.7599<br>AIC= 361,717.9

Supplementary data to '*Assessing the severity of CVD in 213,088 patients with CHD*'- *Zghebi et al. 2020*

**+5 yrs before score**	**Model 4**	• Score 1.23 (1.22; 1.24)<br>• Age 1.11 (1.11; 1.11)<br>• Gender (F) 0.72 (0.71; 0.74)<br>• IMD (Q5) 1.57 (1.52; 1.63)<br>• Race (Asian) 0.92 (0.84; 1.01)<br>**AUROC**=0.7794<br>AIC= 846,039.7	1.20 (1.19; 1.21)<br>**AUROC**=0.7771<br>AIC= 801,618.9	1.19 (1.18; 1.20)<br>**AUROC**= 0.7744<br>AIC= 743,886.8	1.18 (1.17; 1.19)<br>**AUROC**=0.7713<br>AIC=689,968.4	1.16 (1.15; 1.17)<br>**AUROC**=0.7685<br>AIC=637,088.9	1.16 (1.15; 1.18)<br>**AUROC**=0.7655<br>AIC=580,275	1.16 (1.15; 1.18)<br>**AUROC**=0.7627<br>AIC= 525,672.3	1.16 (1.15; 1.18)<br>**AUROC**=0.7602<br>AIC=469,352.6	1.17 (1.15; 1.18)<br>**AUROC**=0.7583<br>AIC=415,256.6	• Score 1.16 (1.15; 1.17)<br>• Age 1.11 (1.10; 1.11)<br>• Gender (F) 0.71 (0.69; 0.73)<br>• IMD (Q5) 1.54 (1.47; 1.62)<br>• Race (Asian) 0.80 (0.68; 0.93)<br>**AUROC**=0.7561<br>AIC=362,395.7

Models were limited to patients contributing to each post-index window

AIC: Akaike information criterion; **AUROC**: area under a Receiver Operating Characteristics; **IMD**: index for multiple deprivation.

Q5: most deprived IMD quintile vs. least deprived IMD quintile. Race: Asian vs. White.

Zghebi SS, *et al. Open Heart* 2021; 8:e001498. doi: 10.1136/openhrt-2020-001498

Supplementary data to '*Assessing the severity of CVD in 213,088 patients with CHD*'- *Zghebi et al. 2020*

Table S6 Cox regression models using moving CV severity scores for CV/DM-related mortality HR (95% CI) – for 2-9 yrs windows models only adjusted HRs for severity scores are presented for simplicity – training dataset

CV/DM death		Index+1 year	Index+2 years	Index+3 years	Index+4 years	Index+5 years	Index+6 years	Index+7 years	Index+8 years	Index+9 years	Index+10 years
**Age Gender IMD, ethnicity Only**	**Model 1**	**AUROC**=0.7844<br>AIC=219,806.1	**AUROC**= 0.7823<br>AIC= 205,935.6	**AUROC**=0.7789<br>AIC= 190,182	**AUROC**=0.7759<br>AIC=176,601.2	**AUROC**=0.7739<br>AIC=163,333	**AUROC**=0.7711<br>AIC= 149,588.6	**AUROC**=0.7686<br>AIC= 135,792.3	**AUROC**= 0.7657<br>AIC= 121,937.1	**AUROC**=0.7624<br>AIC=107,696.1	**AUROC**=0.7600<br>AIC= 93,469.8
**+Ever before score**	**Model 2**	• Score<br>1.50 (1.48; 1.52)<br>• Age<br>1.12 (1.11; 1.12)<br>• Gender (F)<br>0.77 (0.74; 0.80)<br>• IMD (Q5)<br>1.55 (1.45; 1.65)<br>• Race (Asian)<br>1.02 (0.86; 1.22)<br>**AUROC**= 0.7984<br>AIC= 216,823.2	1.47 (1.45; 1.49)<br>**AUROC**=0.7974<br>AIC=203,092	1.45 (1.43; 1.47)<br>**AUROC**=0.7945<br>AIC= 187,508.5	1.44 (1.43; 1.46)<br>**AUROC**=0.7921<br>AIC=174,047.4	1.43 (1.41; 1.45)<br>**AUROC**=0.7901<br>AIC= 160,964.5	1.43 (1.41; 1.45)<br>**AUROC**=0.7879<br>AIC= 147,333	1.42 (1.40; 1.44)<br>**AUROC**=0.7857<br>AIC=133,724.7	1.42 (1.40; 1.44)<br>**AUROC**=0.7840<br>AIC=119,953.9	1.43 (1.41; 1.45)<br>**AUROC**=0.7824<br>AIC=105,807.8	• Score<br>1.42 (1.40; 1.44)<br>• Age<br>1.11 (1.10; 1.11)<br>• Gender (F)<br>0.83 (0.78; 0.88)<br>• IMD (Q5)<br>1.40 (1.27; 1.54)<br>• Race (Asian)<br>0.71 (0.52; 0.97)<br>**AUROC**=0.7803<br>AIC= 918,30.52
**+10 yrs before score**	**Model 3**	• Score<br>1.48 (1.46; 1.50)<br>• Age<br>1.12 (1.11; 1.12)<br>• Gender (F)<br>0.76 (0.73; 0.79)<br>• IMD (Q5)<br>1.54 (1.44; 1.64)<br>• Race (Asian)<br>1.05 (0.88; 1.25)<br>**AUROC**=0.7968<br>AIC= 217,291.4	1.44 (1.42; 1.46)<br>**AUROC**=0.7954<br>AIC= 203,554.9	1.43 (1.41; 1.45)<br>**AUROC**=0.7924<br>AIC= 187,954.1	1.42 (1.40; 1.44)<br>**AUROC**=0.7899<br>AIC= 174,460.1	1.40 (1.38; 1.42)<br>**AUROC**=0.7876<br>AIC= 161,387.1	1.39 (1.38; 1.42)<br>**AUROC**=0.7853<br>AIC= 147,746.5	1.39 (1.37; 1.41)<br>**AUROC**=0.7828<br>AIC= 134,101.6	1.39 (1.37; 1.41)<br>**AUROC**=0.7810<br>AIC=120,299.3	1.40 (1.38; 1.42)<br>**AUROC**=0.7789<br>AIC=106,162.1	• Score<br>1.38 (1.36; 1.41)<br>• Age<br>1.11 (1.11; 1.11)<br>• Gender (F)<br>0.81 (0.77; 0.86)<br>• IMD (Q5)<br>1.40 (1.27; 1.54)<br>• Race (Asian)<br>0.74 (0.54; 1.01)<br>**AUROC**=0.7764<br>AIC= 92,174.03

Supplementary data to '*Assessing the severity of CVD in 213,088 patients with CHD'- Zghebi et al. 2020*

**+5 yrs before score**	**Model 4**	• Score 1.44 (1.41; 1.46)<br>• Age 1.12 (1.12; 1.12)<br>• Gender (F) 0.75 (0.72; 0.78)<br>• IMD (Q5) 1.54 (1.44; 1.64)<br>• Race (Asian) 1.08 (0.90; 1.28)<br>**AUROC**=0.7942<br>AIC= 217,938.5	1.40 (1.38; 1.42)<br>**AUROC**=0.7924<br>AIC= 204,191.6	1.38 (1.36; 1.40)<br>**AUROC**=0.7892<br>AIC= 188,572.5	1.37 (1.35; 1.39)<br>**AUROC**=0.7863<br>AIC=175,067.9	1.35 (1.33; 1.37)<br>**AUROC**=0.7837<br>AIC=161,964.9	1.35 (1.33; 1.38)<br>**AUROC**=0.7793<br>AIC= 148,485.2	1.36 (1.33; 1.38)<br>**AUROC**=0.7761<br>AIC=134,828.3	1.37 (1.35; 1.40)<br>**AUROC**=0.7738<br>AIC=120,991	1.37 (1.35; 1.40)<br>**AUROC**=0.7709<br>AIC=106,834.5	• Score 1.36 (1.34; 1.39)<br>• Age 1.11 (1.11; 1.12)<br>• Gender (F) 0.73 (0.69; 0.78)<br>• IMD (Q5) 1.43 (1.30; 1.57)<br>• Race (Asian) 0.82 (0.61; 1.12)<br>**AUROC**=0.7685<br>AIC= 92,737.82

Models were limited to patients contributing to each post-index window.

AIC: Akaike information criterion; **AUROC**: area under a Receiver Operating Characteristics; **IMD**: index for multiple deprivation.

Q5: most deprived IMD quintile vs. least deprived IMD quintile. Race: Asian vs. White.

Zghebi SS, *et al. Open Heart* 2021; 8:e001498. doi: 10.1136/openhrt-2020-001498

Supplementary data to '*Assessing the severity of CVD in 213,088 patients with CHD'- Zghebi et al. 2020*

Table S7 Multiple events Cox regression models using baseline CV severity scores for any cause hospital admissions HR (95% CI) using Breslow option – training dataset*

Any hosp_breslow Multiple failure	Predictor(s)	HR (95% CI)	AIC
**Model 1 w/o severity score**	**Age**	1.03 (1.02; 1.03)	2.27e+07
	**Gender (F)**	0.80 (0.77; 0.83)	
	**Patient-level IMD**		
	• Q1 (least deprived)	Referent	
	• Q5 (most deprived)	1.37 (1.29; 1.46)	
	• Unknown	0.98 (0.79; 1.22)	
	**Ethnicity**		
	• White	Referent	
	• Asian	1.56 (1.25; 1.94)	
**Model 2**	**Ever before score**	1.3.1 (1.28; 1.34)	2.26e+07
	**Age**	1.02 (1.02; 1.02)	
	**Gender (F)**	0.84 (0.81; 0.87)	
	**Patient-level IMD**		
	• Q1 (least deprived)	Referent	
	• Q5 (most deprived)	1.33 (1.25; 1.42)	
	• Unknown	1.00 (0.80; 1.25)	
	**Ethnicity**		
	• White	Referent	
	• Asian	1.45 (1.17; 1.79)	
**Model 3**	**10 yrs before score**	1.31 (1.27; 1.34)	
	**Age**	1.01 (1.02; 1.02)	2.26e+07
	**Gender (F)**	0.83 (0.80; 0.87)	
	**Patient-level IMD**		
	• Q1 (least deprived)	Referent	
	• Q5 (most deprived)	1.33 (1.25; 1.42)	
	• Unknown	0.99 (0.79; 1.23)	
	**Ethnicity**		
	• White	Referent	
	• Asian	1.46 (1.18; 1.81)	
**Model 4**	**5 yrs before score**	1.31 (1.27; 1.34)	2.27e+07
	**Age**	1.02 (1.02; 1.02)	
	**Gender (F)**	0.83 (0.80; 0.86)	
	**Patient-level IMD**		
	• Q1 (least deprived)	Referent	
	• Q5 (most deprived)	1.34 (1.25; 1.42)	
	• Unknown	0.97 (0.79; 1.22)	
	**Ethnicity**		
	• White	Referent	
	• Asian	1.48 (1.20; 1.84)	

*based on using the longest hospital stay in case of multiple same-day admissions

Supplementary data to '*Assessing the severity of CVD in 213,088 patients with CHD*'- *Zghebi et al. 2020*

Table S8 Poisson regression models using moving severity scores vs. the count of <u>any cause hospitalisation</u> admission(s) in the following year (IRR (95% CI)) - for 2-9 yrs windows models only IRRs for severity scores are presented for simplicity – training dataset

Any apc hosp		**Index+1 year**	**Index+2 years**	**Index+3 years**	**Index+4 years**	**Index+5 years**	**Index+6 years**	**Index+7 years**	**Index+8 years**	**Index+9 years**	**Index+10 years**
**Age Gender IMD, ethnicity Only**	**Model 1**	**AIC**=477,990.9	**AIC**=430,060.8	**AIC**=412,502	**AIC**=399,811.4	**AIC**=390,006.7	**AIC**=383,434.3	**AIC**=369,291.8	**AIC**=345,273.2	**AIC**=325,745.9	**AIC**=309,306
**+Ever before score**	**Model 2**	• Score<br>1.38 (1.38; 1.39)<br>• Age<br>1.01 (1.01; 1.01)<br>• Gender (F)<br>0.84 (0.83; 0.85)<br>• IMD (Q5)<br>1.30 (1.27; 1.33)<br>**AIC**=456,825	1.23 (1.22; 1.23)<br>**AIC**=221,331.4	1.17 (1.16; 1.18)<br>**AIC**=144,601.4	1.13 (1.12; 1.15)<br>**AIC**=115,227.6	1.13 (1.12; 1.14)<br>**AIC**=94,969.21	1.15 (1.14; 1.17)<br>**AIC**=81,186.23	1.17 (1.15; 1.19)<br>**AIC**=65,785.7	1.20 (1.18; 1.22)<br>**AIC**=55,033.83	1.25 (1.23; 1.27)<br>**AIC**=45,422.61	• Score<br>1.19 (1.16; 1.21)<br>• Age<br>1.01 (1.00; 1.01)<br>• Gender (F)<br>1.13 (1.07; 1.19)<br>• IMD (Q5)<br>1.07 (0.97; 1.17)<br>**AIC**=37,918.6
**+10 yrs before score**	**Model 3**	• Score<br>1.39 (1.39; 1.40)<br>• Age<br>1.01 (1.01; 1.01)<br>• Gender (F)<br>0.84 (0.83; 0.85)<br>• IMD (Q5)<br>1.30 (1.27; 1.32)<br>**AIC**=457,753.5	1.23 (1.22; 1.24)<br>**AIC**=221,537.4	1.18 (1.17; 1.19)<br>**AIC**=144,624.4	1.14 (1.13; 1.16)<br>**AIC**=115,206.7	1.15 (1.13; 1.16)<br>**AIC**=94,920.38	1.18 (1.17; 1.20)<br>**AIC**=81,078.77	1.22 (1.20; 1.24)<br>**AIC**=65,587.48	1.28 (1.26; 1.30)<br>**AIC**=54,718.66	**1.38 (1.35; 1.40)**<br>**AIC= 44,921.51**	• Score<br>1.37 (1.34; 1.40)<br>• Age<br>1.01 (1.00;1.01)<br>• Gender (F)<br>1.15 (1.09; 1.22)<br>• IMD (Q5)<br>1.04 (0.95; 1.14)<br>AIC=37,389.42
**+5 yrs before score**	**Model 4**	• Score<br>1.40 (1.39; 1.41)<br>• Age<br>1.01 (1.01; 1.01)<br>• Gender (F)<br>0.83 (0.82; 0.84)<br>• IMD (Q5)<br>1.31 (1.28; 1.33)<br>**AIC**=460,109	1.22 (1.21; 1.23)<br>**AIC**=221,985.3	1.18 (1.17; 1.19)<br>**AIC**=144,758.2	1.14 (1.13; 1.16)<br>**AIC**=115,260.8	1.17 (1.15; 1.19)<br>**AIC**=94,855.22	1.32 (1.30; 1.34)<br>AIC=80,588.38	1.47 (1.44; 1.49)<br>**AIC**=64,630.33	1.57 (1.54; 1.60)<br>**AIC**=53,757.77	1.72 (1.69; 1.76)<br>**AIC**=43,891.78	• Score<br>1.71 (1.66; 1.75)<br>• Age<br>1.01 (1.01; 1.12)<br>• Gender (F)<br>1.06 (1.00; 1.17)<br>• IMD (Q5)<br>1.03 (0.94; 1.13)<br>AIC=36,749.45

Models were limited to patients contributing to each post-index window.

AIC: Akaike information criterion; **AUROC**: area under a Receiver Operating Characteristics; **IMD**: index for multiple deprivation.

Q5: most deprived IMD quintile vs. least deprived IMD quintile. Race: Asian vs. White.

Supplementary data to '*Assessing the severity of CVD in 213,088 patients with CHD'- Zghebi et al. 2020*

Table S9 Survival models for 1, 3, 5, and 10-year aggregated any hospitalisation and all-cause mortality outcome using baseline scores HR (95% CI) – training dataset

any hosp or all-death	Predictor(s)	1-year	3-year	5-year	10-year
**Model 1 w/o severity score**	**Age, gender, deprivation, ethnicity only**	**AUROC**= 0.6055 **AIC**= 1,008,481	**AUROC**= 0.6115 **AIC**= 1,995,512	**AUROC**= 0.6140 **AIC**= 2,424,369	**AUROC**= 0.6169 **AIC**= 2,915,837
**Model 2**	**Ever before score**	1.27 (1.26; 1.28) **AUROC**= 0.6271 **AIC**= 1,004,865	1.24 (1.23; 1.25) **AUROC**= 0.6288 **AIC**= 1,990,204	1.22 (1.22; 1.23) **AUROC**= 0.6294 **AIC**= 2,418,915	1.21 (1.20; 1.21) **AUROC**= 0.6307 **AIC**= 2,910,327
	**Age**	1.01 (1.01; 1.01)	1.02 (1.02; 1.02)	1.02 (1.02; 1.02)	1.02 (1.02; 1.02)
	**Gender (F)**	0.72 (0.70; 0.73)	0.80 (0.79; 0.81)	0.83 (0.82; 0.84)	0.85 (0.84; 0.86)
	**IMD (vs. least deprived)**				
	• Q5 (most deprived)	1.05 (1.02; 1.09)	1.14 (1.12; 1.17)	1.17 (1.14; 1.19)	1.18 (1.16; 1.20)
	**Ethnicity (vs. White)**				
	• Black	0.89 (0.80; 0.98)	0.97 (0.90; 1.04)	1.01 (0.95; 1.08)	1.02 (0.96; 1.08)
	• Asian	1.33 (1.26; 1.41)	1.28 (1.23; 1.33)	1.26 (1.21; 1.31)	1.23 (1.19; 1.28)
	• Mixed	0.96 (0.80; 1.15)	1.02 (0.90; 1.16)	1.08 (0.96; 1.21)	1.02 (0.92; 1.14)
	• Other	1.02 (0.92; 1.14)	0.98 (0.91; 1.06)	0.98 (0.91; 1.05)	0.95 (0.89; 1.02)
	• Unknown	0.26 (0.24; 0.28)	0.25 (0.24; 0.26)	0.25 (0.24; 0.26)	0.26 (0.25; 0.26)
**Model 3**	**10 yrs before score**	1.28 (1.27; 1.29) **AUROC**= 0.6270 **AIC**= 1,004,939	1.24 (1.24; 1.25) **AUROC**= 0.6281 **AIC**= 1,990,505	1.22 (1.22; 1.23) **AUROC**= 0.6286 **AIC**= 2,419,291	1.21 (1.20; 1.21) **AUROC**= 0.6296 **AIC**= 2,910,771
	**Age**	1.01 (1.01; 1.01)	1.02 (1.02; 1.02)	1.02 (1.02; 1.02)	1.02 (1.02; 1.02)
	**Gender (F)**	0.72 (0.70; 0.73)	0.80 (0.79; 0.81)	0.83 (0.82; 0.84)	0.84 (0.84; 0.85)
	**IMD (vs. least deprived)**				
	• Q5 (most deprived)	1.05 (1.02; 1.09)	1.14 (1.12; 1.17)	1.16 (1.14; 1.19)	1.18 (1.16; 1.20)
	**Ethnicity (vs. White)**				
	• Black	0.89 (0.80; 0.99)	0.98 (0.91; 1.05)	1.02 (0.96; 1.09)	1.03 (0.97; 1.09)
	• Asian	1.34 (1.27; 1.42)	1.29 (1.24; 1.34)	1.27 (1.22; 1.32)	1.24 (1.19; 1.29)
	• Mixed	0.96 (0.80; 1.16)	1.02 (0.90; 1.16)	1.08 (0.96; 1.21)	1.02 (0.92; 1.14)
	• Other	1.02 (0.92; 1.14)	0.99 (0.91; 1.07)	0.98 (0.92; 1.06)	0.95 (0.89; 1.02)
	• Unknown	0.26 (0.24; 0.28)	0.25 (0.24; 0.26)	0.25 (0.24; 0.26)	0.26 (0.25; 0.26)
**Model 4**	**5 yrs before score**	1.30 (1.29; 1.31) **AUROC**= 0.6265 **AIC**= 1,005,101	1.25 (1.24; 1.26) **AUROC**= 0.6266 **AIC**= 1,991,076	1.23 (1.22; 1.23) **AUROC**= 0.6268 **AIC**= 2,419,997	1.21 (1.20; 1.21) **AUROC**=0.6277 **AIC**= 2,911,543
	**Age**	1.01 (1.01; 1.01)	1.02 (1.02; 1.02)	1.02 (1.02; 1.02)	1.02 (1.02; 1.02)
	**Gender (F)**	0.71 (0.70; 0.73)	0.79 (0.78; 0.80)	0.82 (0.81; 0.83)	0.84 (0.83; 0.85)
	**IMD (vs. least deprived)**				
	• Q5 (most deprived)	1.05 (1.02; 1.09)	1.14 (1.12; 1.17)	1.17 (1.14; 1.19)	1.18 (1.16; 1.20)
	**Ethnicity (vs. White)**				
	• Black	0.90 (0.81; 0.99)	0.98 (0.92; 1.06)	1.03 (0.97; 1.10)	1.04 (0.98; 1.10)
	• Asian	1.36 (1.29; 1.44)	1.31 (1.25; 1.36)	1.28 (1.23; 1.34)	1.25 (1.21; 1.30)
	• Mixed	0.97 (0.81; 1.16)	1.03 (0.90; 1.17)	1.08 (1.00; 1.21)	1.02 (0.92; 1.14)
	• Other	1.03 (0.92; 1.14)	0.99 (0.92; 1.07)	0.99 (0.92; 1.06)	0.96 (0.90; 1.02)
	• Unknown	0.26 (0.24; 0.28)	0.25 (0.24; 0.28)	0.25 (0.24; 0.26)	0.25 (0.25; 0.26)

Supplementary data to '*Assessing the severity of CVD in 213,088 patients with CHD'- Zghebi et al. 2020*

Table S10 - Summary of AUROCs of fitted severity score-only Cox regression models – training and validation datasets

AUROC (Gonen and Heller's K)	**Training dataset**				**Validation dataset**			
Outcome	**Ever before score (Unlimited)**	**10-year before score**	**5-year before score**	**Unlimited score categories**	**Ever before score (Unlimited)**	**10-year before score**	**5-year before score**	**Unlimited score categories**
**1-year all-cause mortality**	0.6446	0.6367	0.6241	0.6391	0.6445	0.6333	0.6230	0.6307
**3-year all-cause mortality**	0.6424	0.6333	0.6188	0.6335	0.6399	0.6311	0.6442	0.6203
**5-year all-cause mortality**	0.6380	0.6288	0.6146	0.6276	0.6406	0.6315	0.6172	0.6269
**10-year all-cause mortality**	0.6646	0.6203	0.6054	0.6185	0.6340	0.6248	0.6092	0.6194
**1-year CV/diabetes mortality**	0.6662	0.6584	0.6429	0.6538	0.6617	0.6564	0.6416	0.6383
**3-year CV/diabetes mortality**	0.6669	0.6582	0.6430	0.6645	0.6671	0.6602	0.6452	0.6347
**5-year CV/diabetes mortality**	0.6646	0.6558	0.6413	0.6599	0.6690	0.6609	0.6453	0.6448
**10-year CV/diabetes mortality**	0.6598	0.6501	0.6345	0.6518	0.6616	0.6540	0.6376	0.6412
**1-year aggregated any hospitalisation or mortality**	0.5845	0.5832	0.5801	0.5809	-	-	-	-
**3-year aggregated any hospitalisation or mortality**	0.5829	0.5801	0.5746	0.5747	-	-	-	-
**5-year aggregated any hospitalisation or mortality**	0.5812	0.5779	0.5715	0.5718	-	-	-	-
**10-year aggregated any hospitalisation or mortality**	0.5799	0.5760	0.5692	0.5695	-	-	-	-

Supplementary data to '*Assessing the severity of CVD in 213,088 patients with CHD'- Zghebi et al. 2020*

Table S11 f of AIC and AUROCs of fitted Cox regression models without IMD – training dataset

Model	1-year all-cause mortality	3-year all-cause mortality	5-year all-cause mortality	10-year all-cause mortality	1-year CV/diabetes-related mortality	3-year CV/diabetes-related mortality	5-year CV/diabetes-related mortality	10-year CV/diabetes-related mortality	1-year aggregated any hospitalisation or mortality	3-year aggregated any hospitalisation or mortality	5-year aggregated any hospitalisation or mortality	10-year aggregated any hospitalisation or mortality
**Model 1a**	AUROC=0.7860 (AIC=43,059)	AUROC=0.7821 (AIC=155,632.1)	AUROC=0.7808 (AIC= 265,700.5)	AUROC=0.7776 (AIC= 533,430.3)	AUROC=0.7958 (AIC=13,382)	AUROC=0.7955 (AIC=42,933.69)	AUROC=0.7930 (AIC= 69,906.25)	AUROC=0.7899 (AIC= 138,197)	AUROC=0.6051 (AIC= 1,008,511)	AUROC=0.6107 (AIC=1,995,732)	AUROC=0.6131 (AIC= 2,424,698)	AUROC=0.6160 (AIC=2,916,288)
**Model 2a**	AUROC= 0.7906 (AIC=42,597.87)	AUROC=0.7875 (AIC= 154,095.5)	AUROC=0.7866 (AIC= 263,462)	AUROC=0.7843 (AIC=530,087.5)	AUROC= 0.8024 (AIC= 13,091.68)	AUROC=0.8033 (AIC= 42,008.19)	AUROC=0.8016 (AIC=68,518.67)	AUROC= 0.8004 (AIC= 135,915.5)	AUROC=0.6269 (AIC= 1,004,876)	AUROC=0.6283 (AIC=1,990,366)	AUROC=0.6288 (AIC= 2,419,175)	AUROC=0.6301 (AIC=2,910,691)
**Model 3a**	AUROC= 0.7907 (AIC=42,659.83)	AUROC=0.7872 (AIC= 154,364.7)	AUROC=0.7861 (AIC=263,843.8)	AUROC=0.7836 (AIC= 530,648.4)	AUROC=0.8026 (AIC=13,129)	AUROC=0.8031 (AIC= 42,150.3)	AUROC=0.8013 (AIC= 68,725.07)	AUROC=0.7995 (AIC= 136,260.7)	AUROC=1004949 (AIC= 1,004,949)	AUROC=0.6276 (AIC=1,990,664)	AUROC=0.6280 (AIC=2,419,548)	AUROC=0.6290 (AIC=2,911,132)
**Model 4a**	AUROC= 0.7905 (AIC=42,742.73)	AUROC=0.7866 (AIC= 154,696.2)	AUROC=0.7854 (AIC=264,316.9)	AUROC=0.7824 (AIC=531,402.4)	AUROC= 0.8018 (AIC= 13,199.54)	AUROC=0.8023 (AIC=42,357.11)	AUROC=0.8003 (AIC=69,012.6)	AUROC=0.7978 (AIC=136,754.8)	AUROC=0.6263 (AIC= 1,005,112)	AUROC=0.6261 (AIC= 1,991,239)	AUROC=0.6262 (AIC=2,420,258)	AUROC=0.6271 (AIC=2,911,909)

*Competing risk analysis; $^{\$}$ adjusted for age, gender, IMD only.
Model 1a: age, gender, and ethnicity.
Model 2a: Model 1a + ever before severity score.
Model 3a: Model 1a + 10-year severity score
Model 4a: Model 1a + 5-year severity score
AIC: Akaike information criterion; **AUROC**: area under a Receiver Operating Characteristics curve; **CV**: cardiovascular.

Supplementary data to '*Assessing the severity of CVD in 213,088 patients with CHD'- Zghebi et al. 2020*

Table S12 Calibration test results for baseline CVD scores in training and validation datasets using Somer's D

Somer's D	Training dataset				Validation dataset			
	**Ever before score (Unlimited)**	**10-year before score**	**5-year before score**	**Unlimited score categories**	**Ever before score (Unlimited)**	**10-year before score**	**5-year before score**	**Unlimited score categories**
**1-year all-cause mortality**	0.2891	0.2734	0.2482	0.2782	0.2889	0.2667	0.2459	0.2615
**3-year all-cause mortality**	0.2848	0.2665	0.2376	0.2671	0.2799	0.2622	0.2349	0.2407
**5-year all-cause mortality**	0.2760	0.2576	0.2292	0.2552	0.2812	0.2631	0.2343	0.2537
**10-year all-cause mortality**	0.2600	0.2407	0.2109	0.2369	0.2680	0.2496	0.2185	0.2389
**1-year CV/diabetes mortality**	0.3323	0.3169	0.2858	0.3077	0.3233	0.3128	0.2832	0.2765
**3-year CV/diabetes mortality**	0.3338	0.3165	0.2861	0.3290	0.3341	0.3205	0.2904	0.2694
**5-year CV/diabetes mortality**	0.3293	0.3116	0.2827	0.3198	0.3380	0.3218	0.2905	0.2895
**10-year CV/diabetes mortality**	0.3196	0.3003	0.2690	0.3035	0.3232	0.3080	0.2752	0.2823
**1-year aggregated any hospitalisation or mortality**	0.1691	0.1664	0.1601	0.1618	-	-	-	-
**3-year aggregated any hospitalisation or mortality**	0.1658	0.1850	0.1491	0.1495	-	-	-	-
**5-year aggregated any hospitalisation or mortality**	0.1624	0.1558	0.1431	0.1436	-	-	-	-
**10-year aggregated any hospitalisation or mortality**	0.1598	0.1521	0.1385	0.1390	-	-	-	-

Supplementary data to '*Assessing the severity of CVD in 213,088 patients with CHD'- Zghebi et al. 2020*

Table S13 Calibration test results for moving CVD scores in training dataset using Somer's D

Somer's D		Index+1 year	Index+2 years	Index+3 years	Index+4 years	Index+5 years	Index+6 years	Index+7 years	Index+8 years	Index+9 years	Index+10 years
**All-cause mortality**	**Unlimited score**	0.2393	0.2324	0.2280	0.2253	0.2234	0.2246	0.2241	0.2272	0.2295	0.2305
	**10-year before score**	0.2176	0.2098	0.2064	0.2037	0.2010	0.2022	0.2014	0.2055	0.2071	0.2054
	**5-year before score**	0.1866	0.1784	0.1744	0.1708	0.1651	0.1492	0.1437	0.1423	0.1396	0.1374
**CV/diabetes mortality**	**Unlimited score**	0.3081	0.3064	0.3054	0.3066	0.3054	0.3075	0.3077	0.3128	0.3186	0.3168
	**10-year before score**	0.2857	0.2829	0.2822	0.2837	0.2807	0.2823	0.2825	0.2889	0.2926	0.2878
	**5-year before score**	0.2514	0.2476	0.2458	0.2461	0.2407	0.2161	0.2093	0.2112	0.2088	0.2047

Supplementary data to '*Assessing the severity of CVD in 213,088 patients with CHD'- Zghebi et al. 2020*

Table S14 Survival models for 1-year all-cause mortality outcome using baseline scores HR (95% CI) - Validation dataset

1-yr all-cause death	Predictor(s)	HR (95% CI)	AUROC AIC
**Model 1 w/o severity score**	**Age, gender, deprivation, ethnicity only**	HRs Similar to below	**AUROC**= 0.7862 **AIC**= 10,023.61
**Model 2**	**Ever before score**	1.40 (1.32 to 1.48)	**AUROC**= 0.7908 **AIC**= 9,903.685
	**Age**	1.11 (1.10 to 1.12)	
	**Gender (F)**	0.64 (0.54 to 0.76)	
	**IMD (vs. least deprived)**		
	• Q5 (most deprived)	1.33 (1.00 to 1.76)	
	**Ethnicity (vs. White)**		
	• Black	1.03 (0.33 to 3.20)	
	• Asian	1.63 (0.89 to 2.98)	
	• Mixed	3.44 (0.86 to 13.8)	
	• Other	0.62 (0.15 to 2.47)	
	• Unknown	1.37 (0.94 to 1.99)	
**Model 3**	**10 yrs before score**	1.36 (1.28 to 1.45)	**AUROC**= 0.7899 **AIC**= 9,935.482
	**Age**	1.11 (1.10 to 1.12)	
	**Gender (F)**	0.64 (0.53 to 0.76)	
	**IMD (vs. least deprived)**	Referent	
	• Q5 (most deprived)	1.32 (1.00 to 1.76)	
	**Ethnicity (vs. White)**		
	• Black	1.08 (0.35 to 3.37)	
	• Asian	1.66 (0.91 to 3.04)	
	• Mixed	3.34 (0.83 to 13.4)	
	• Other	0.64 (0.16 to 2.58)	
	• Unknown	1.31 (0.90 to 1.91)	
**Model 4**	**5 yrs before score**	1.38 (1.29 to 1.47)	**AUROC=** 0.7901 **AIC**= 9,946.772
	**Age**	1.12 (1.11 to 1.13)	
	**Gender (F)**	0.63 (0.53 to 0.75)	
	**IMD (vs. least deprived)**		
	Q5 (most deprived)	1.30 (0.98 to 1.73)	
	**Ethnicity (vs. White)**		
	• Black	1.09 (0.35 to 3.41)	
	• Asian	1.71 (0.94 to 3.11)	
	• Mixed	3.44 (0.86 to 13.8)	
	• Other	0.67 (0.17 to 2.71)	
	• Unknown	1.28 (0.88 to 1.86)	

Supplementary data to '*Assessing the severity of CVD in 213,088 patients with CHD*'- *Zghebi et al. 2020*

Table S15 Survival models for 1, 3, 5, and 10-year CV/diabetes-related mortality outcome using baseline scores HR (95% CI) - Validation dataset

1-10yr CV/DM death	Predictor(s)	1-year	3-year	5-year	10-year
**Model 1 w/o severity score**	**Age, gender, deprivation, ethnicity only**	HR Similar to below (**AUROC**= 0.7934 (**AIC**= 3,295.584	HR Similar to below (**AUROC**= 0.7945) (**AIC**= 9,555.281)	HR Similar to below (**AUROC**= 0.7869) (**AIC**= 15,109.95)	HR Similar to below (**AUROC**= 0.7852) (**AIC**= 29,798.48)
**Model 2**	**Ever before score**	1.54 (1.40 to 1.69) **AUROC**=0.7993 **AIC**=3,227.852	1.59 (1.51 to 1.68) **AUROC**= 0.8018 **AIC**= 9,324.133	1.62 (1.55 to 1.70) **AUROC**= 0.7958 **AIC**= 14,734.48	1.57 (1.52 to 1.63) **AUROC**= 0.7959 **AIC**= 29,239.54
	**Age**	1.11 (1.10 to 1.13)	1.12 (1.10 to 1.13)	1.11 (1.10 to 1.12)	1.11 (1.10 to 1.12)
	**Gender (F)**	0.63 (0.47 to 0.86)	0.78 (0.65 to 0.93)	0.78 (0.67 to 0.90)	0.73 (0.66 to 0.81)
	**IMD (vs. least deprived)** • Q5 (most deprived)	1.21 (0.72 to 2.04)	1.27 (0.94 to 1.72)	1.28 (1.00 to 1.63)	1.37 (1.16 to 1.63)
	**Ethnicity (vs. White)**				
	• Black	2.11 (0.52 to 8.58)	1.55 (0.58 to 4.16)	2.16 (1.11 to 4.18)	1.30 (0.70 to 2.43)
	• Asian	0.44 (0.06 to 3.15)	0.64 (0.24 to 1.72)	0.98 (0.52 to 1.83)	0.79 (0.48 to 1.30)
	• Mixed	5.24 (0.73 to 37.5)	3.58 (0.89 to 14.4)	2.20 (0.55 to 8.84)	1.46 (0.47 to 4.21)
	• Other	0.91 (0.13 to 6.53)	1.26 (0.47 to 3.37)	1.38 (0.66 to 2.91)	0.83 (0.41 to 1.66)
	• Unknown	1.52 (0.80 to 2.90)	1.45 (0.98 to 2.14)	1.50 (1.10 to 2.03)	1.44 (1.16 to 1.78)
**Model 3**	**10 yrs before score**	1.55 (1.41 to 1.71) **AUROC**= 0.7989 **AIC**= 3,233.783	1.59 (1.50 to 1.69) (**AUROC**= 0.8016) **AIC**= 9,353.166	1.61 (1.53 to 1.69) **AUROC**= 0.7952 **AIC**=14,792.79	1.57 (1.51 to 1.63) **AUROC**=0.7949) **AIC=**29304.99
	**Age**	1.12 (1.10 to 1.13)	1.12 (1.11 to 1.13)	1.11 (0.67 to 0.89)	1.11 (1.11 to 1.12)
	**Gender (F)**	0.63 (0.46 to 0.86)	0.77 (0.64 to 0.93)	0.77 (0.70 to 0.81)	0.73 (0.66 to 0.80)
	**IMD (vs. least deprived)**	Referent	Referent	Referent	Referent
	• Q5 (most deprived)	1.21 (0.72 to 2.04)	1.28 (0.95 to 1.73)	1.28 (1.01 to 1.64)	1.37 (1.16 to 1.63)
	**Ethnicity (vs. White)**				
	• Black	2.30 (0.57 to 9.35)	1.69 (0.63 to 2.65)	2.04 (1.24 to 4.67)	1.43 (0.77 to 2.68)
	• Asian	0.44 (0.06 to 3.18)	0.64 (0.24 to 1.72)	0.99 (0.53 to 1.86)	0.80 (0.49 to 1.31)
	• Mixed	5.05 (0.71 to 36.2)	3.39 (0.84 to 13.6)	2.09 (0.52 to 8.36)	1.40 (0.45 to 4.35)
	• Other	0.98 (0.14 to 6.97)	1.35 (0.50 to 3.62)	1.51 (0.72 to 3.18)	0.89 (0.44 to 1.78)
	• Unknown	1.47 (0.77 to 2.79)	1.38 (0.93 to 2.04)	1.43 (1.05 to 1.94)	1.39 (1.12 to 1.72)
**Model 4**	**5 yrs before score**	1.54 (1.38 to 1.71) **AUROC=** 0.7989 **AIC=** 3,247.165	1.58 (1.48 to 1.69) **AUROC**=0.8014 **AIC=** 9,397.813	1.59 (1.51 to 1.68) **AUROC=** 0.7947 **AIC=** 14,863	1.54 (1.48 to 1.61) **AUROC=** 0.7935) **AIC=** 29,416.99
	**Age**	1.12 (1.10 to 1.14)	1.12 (1.11 to 1.13)	1.12 (1.11 to 1.12)	1.12 (1.11 to 1.12)
	**Gender (F)**	0.62 (0.46 to 0.85)	0.76 (0.64 to 0.92)	0.76 (0.66 to 0.88)	0.72 (0.65 to 0.80)
	**IMD (vs. least deprived)** Q5 (most deprived)	1.18 (0.70 to 1.98)	1.27 (0.94 to 1.71)	1.27 (1.00 to 1.62)	1.36 (1.15 to 1.61)
	**Ethnicity (vs. White)**				
	• Black	2.34 (0.58 to 9.52)	1.71 (0.64 to 4.59)	2.43 (1.25 to 4.71)	1.44 (0.77 to 2.68)
	• Asian	0.46 (0.06 to 3.32)	0.67 (0.25 to 1.80)	1.05 (0.56 to 1.960)	0.85 (0.52 to 1.39)
	• Mixed	5.23 (0.73 to 37.8)	3.54 (0.88 to 14.2)	2.14 (0.53 to 8.60)	1.44 (0.46 to 4.48)
	• Other	1.04 (0.15 to 7.47)	1.48 (0.55 to 3.97)	1.66 (0.79 to 3.50)	0.96 (0.48 to 1.92)
	• Unknown	1.39 (0.73 to 2.65)	1.32 (0.89 to 1.94)	1.35 (1.00 to 1.84)	1.34 (1.08 to 1.66)

Supplementary data to '*Assessing the severity of CVD in 213,088 patients with CHD'- Zghebi et al. 2020*

Table S16 Competing risk analysis models for 1-year any cause or CV/DM-related hospitalisation (competed by all-cause death) using baseline scores - Validation dataset

		1-year any cause hospitalisation		1-year CV/DM hospitalisation	
**1-yr hosp (competing)**	**Predictor(s)**	**SHR (95% CI)**	**AIC**	**SHR (95% CI)**	**AIC**
**Model 1 w/o severity score**	**Age, gender, deprivation, ethnicity only**	HR Similar to below	**220,363.5**	HR Similar to below	**156,705.3**
**Model 2**	**Ever before score**	1.26 (1.25; 1.28)	**219,487.8**	1.36 (1.34; 1.38)	**155,549.8**
	**Age**	1.01 (1.01; 1.01)		1.01 (1.00; 1.01)	
	**Gender (F)**	0.71 (0.69; 0.74)		0.60 (0.58; 0.63)	
	**IMD (vs. least deprived)**				
	Q5 (most deprived)	1.07 (1.01; 1.14)		1.04 (0.97; 1.12)	
	**Ethnicity (vs. White)**				
	• Black	0.72 (0.57; 0.90)		0.64 (0.48; 0.84)	
	• Asian	1.13 (1.01; 1.27)		1.30 (0.98; 1.30)	
	• Mixed	1.25 (0.79; 1.14)		1.26 (0.84; 1.91)	
	• Other	0.98 (0.80; 1.21)		0.97 (0.76; 1.25)	
	• Unknown	0.25 (0.22; 0.29)		0.26 (0.22; 0.31)	
**Model 3**	**10 yrs before score**	1.27 (1.25; 1.29)	**219,551**	1.36 (1.34 to 1.39)	**155,634.7**
	**Age**	1.01 (1.01; 1.01)		1.01 (1.01 to 1.01)	
	**Gender (F)**	0.71 (0.69; 0.74)		0.60 (0.58 to 0.63)	
	**IMD (vs. least deprived)**				
	Q5 (most deprived)	1.06 (1.00; 1.13)		1.03 (0.96; 1.11)	
	**Ethnicity (vs. White)**				
	• Black	0.73 (0.59; 0.92)		0.66 (0.50; 0.87)	
	• Asian	1.14 (1.02; 1.28)		1.14 (0.99; 1.31)	
	• Mixed	1.25 (0.87; 1.78)		1.26 (0.83; 1.91)	
	• Other	0.98 (0.80; 1.21)		0.98 (0.76; 1.25)	
	• Unknown	0.25 (0.22; 0.29)		0.26 (0.21; 0.30)	
**Model 4**	**5 yrs before score**	1.29 (1.27; 1.31)	**219,566.1**	1.39 (1.36; 1.42)	**155,676.2**
	**Age**	1.01 (1.01; 1.01)		1.01 (1.01; 1.01)	
	**Gender (F)**	0.71 (0.69; 0.74)		0.60 (0.57; 0.63)	
	**IMD (vs. least deprived)**				
	Q5 (most deprived)	1.06 (1.00; 1.13)		1.03 (0.95; 1.10)	
	**Ethnicity (vs. White)**				
	• Black	0.74 (0.59; 0.93)		0.67 (0.50; 0.89)	
	• Asian	1.16 (1.04; 1.31)		1.17 (1.02; 1.35)	
	• Mixed	1.25 (0.88; 1.79)		1.26 (0.84; 1.91)	
	• Other	1.00 (0.81; 1.23)		1.00 (0.78; 1.28)	
	• Unknown	0.25 (0.22; 0.29)		0.25 (0.21; 0.30)	

Supplementary data to ‘*Assessing the severity of CVD in 213,088 patients with CHD’- Zghebi et al. 2020*

Table S17 Summary of AIC and AUROCs of fitted regression models – Validation dataset

**Predictors /model**	**1-year all-cause mortality**	**1-year CV/diabetes-related mortality**	**1-year hospitalisation (Single event)***	**1-year CV/diabetes-related hospitalisa-tion***
**Demographics-only model**				
**Model 1**	AUROC=0.7862 (AIC= 10,023.61)	AUROC= 0.7934 (AIC= 3,295.584)	AIC= 220,363.5	AIC= 156,705.3
**Severity score + demographics models**				
**Model 2** (Model 1 + ever before severity score)	AUROC= 0.7908 (AIC= 9,903.685)	AUROC= 0.7993 (AIC= 3,227.85)	AIC= 219,487.8	AIC= 155,549.8
**Model 3 (**Model 1 + 10-year severity score)	AUROC= 0.7899 (AIC= 9,935.482)	AUROC= 0.7989 (AIC= 3,233.783)	AIC= 21,9551	AIC= 155,634.7
**Model 4** (Model 1 + 5-year severity score)	AUROC= 0.7901 (AIC= 9,946.772)	AUROC= 0.7989 (AIC= 3,247.165)	AIC= 219,566.1	AIC= 155,676.2

*Competing risk analysis; $^{\$}$ adjusted for age, gender, IMD only

Supplementary data to '*Assessing the severity of CVD in 213,088 patients with CHD*'- *Zghebi et al. 2020*

Table S18 Summary of AIC and AUROCs of fitted Cox regression models without IMD – validation dataset

Predictors /model	1-year all-cause mortality	3-year all-cause mortality	5-year all-cause mortality	10-year all-cause mortality	1-year CV/diabetes-related mortality	3-year CV/diabetes-related mortality	5-year CV/diabetes-related mortality	10-year CV/diabetes-related mortality
**Model 1a**	AUROC= 0.7859 (AIC= 10,025.01)	AUROC= 0.7841 (AIC= 34,719.34)	AUROC= 0.7836 (AIC= 59,255.96)	AUROC= 0.7785 (AIC= 116,868.5)	AUROC= 0.7930 (AIC= 3,291.006)	AUROC= 0.7945 (AIC= 9,551.787)	AUROC= 0.7869 (AIC= 15,110.67)	AUROC= 0.7851 (AIC= 29,815.99)
**Model 2a**	AUROC= 0.7905 (AIC= 9,904.208)	AUROC= 0.7888 (AIC= 34,378.47)	AUROC= 0.7890 (AIC= 58,681.04)	AUROC= 0.7851 (AIC= 115,971.3)	AUROC= 0.7990 (AIC= 3,221.004)	AUROC= 0.8018 (AIC= 9,319.359)	AUROC= 0.7959 (AIC= 14,732.54)	AUROC= 0.7958 (AIC= 29,254.44)
**Model 3a**	AUROC= 0.7896 (AIC= 9,936.146)	AUROC= 0.7884 (AIC= 34,443.99)	AUROC= 0.7883 (AIC= 58,788.02)	AUROC= 0.7842 (AIC= 116,120.1)	AUROC= 0.7986 (AIC= 3,226.968)	AUROC= 0.8015 (AIC= 9,350.691)	AUROC= 0.7953 (AIC= 14,791.14)	AUROC= 0.7948 (AIC= 29,319.57)
**Model 4a**	AUROC= 0.7899 (AIC= 9,946.966)	AUROC= 0.7882 (AIC= 34,501.83)	AUROC= 0.7879 (AIC= 58,889.06)	AUROC= 0.7831 (AIC= 116,304.7)	AUROC= 0.7987 (AIC= 32,42.228)	AUROC=0.8013 (AIC= 9,394.803)	AUROC= 0.7947 (AIC= 14,861.42)	AUROC= 0.7933 (AIC= 29,430.88)

*Competing risk analysis; $ adjusted for age, gender, IMD only.
Model 1a: age, gender, and ethnicity.
Model 2a: Model 1a + ever before severity score.
Model 3a: Model 1a + 10-year severity score
Model 4a: Model 1a + 5-year severity score
AIC: Akaike information criterion; **AUROC**: area under a Receiver Operating Characteristics curve; **CV**: cardiovascular.

Supplementary data to '*Assessing the severity of CVD in 213,088 patients with CHD'- Zghebi et al. 2020*

Figure S1 Scatter plot of estimated AIC and AUROC for models with and without severity scores for three outcomes at 1 year

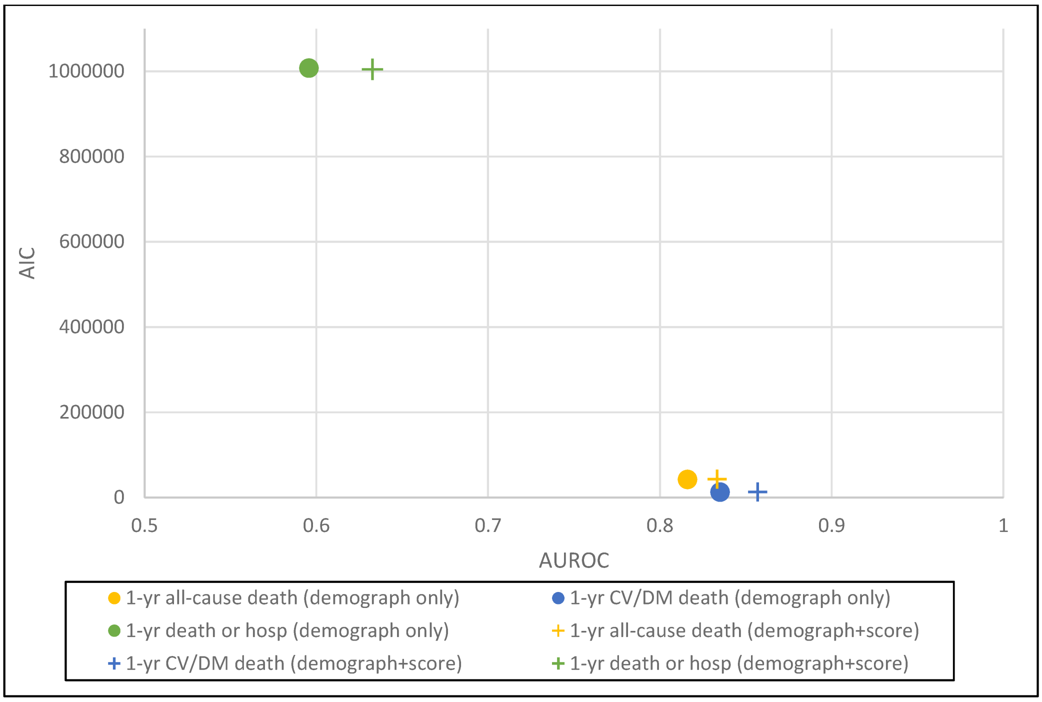

Supplementary data to '*Assessing the severity of CVD in 213,088 patients with CHD*'- *Zghebi et al. 2020*

Figure S2 Kaplan-Meier survivor plots for adverse outcomes by CV severity score categories -Validation data

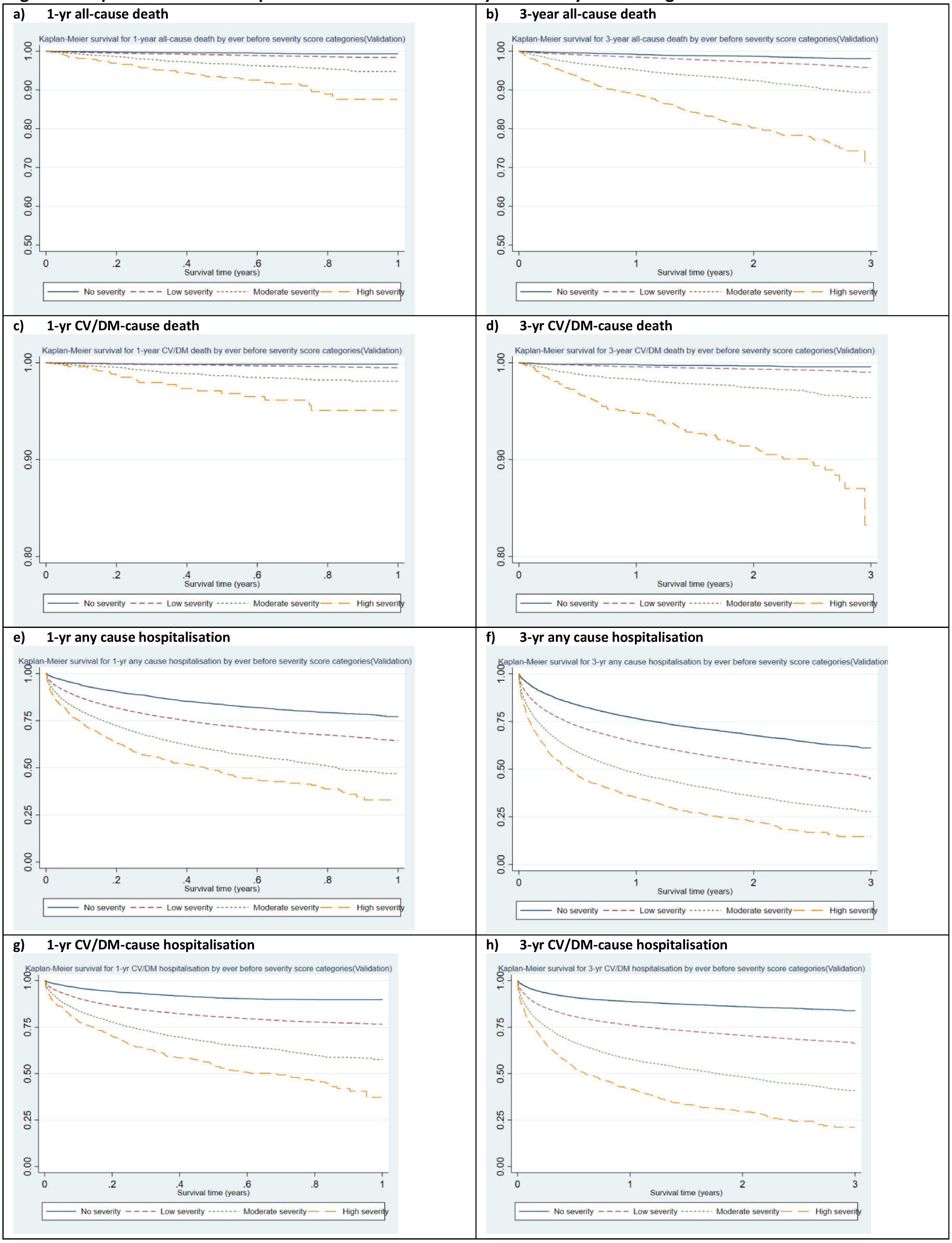

Supplementary data to '*Assessing the severity of CVD in 213,088 patients with CHD'- Zghebi et al. 2020*

Figure S3 Calibration test by prediction of population-averaged survival probabilities for CVD scores for 1-year all-cause mortality (primary outcome) in training and validation datasets

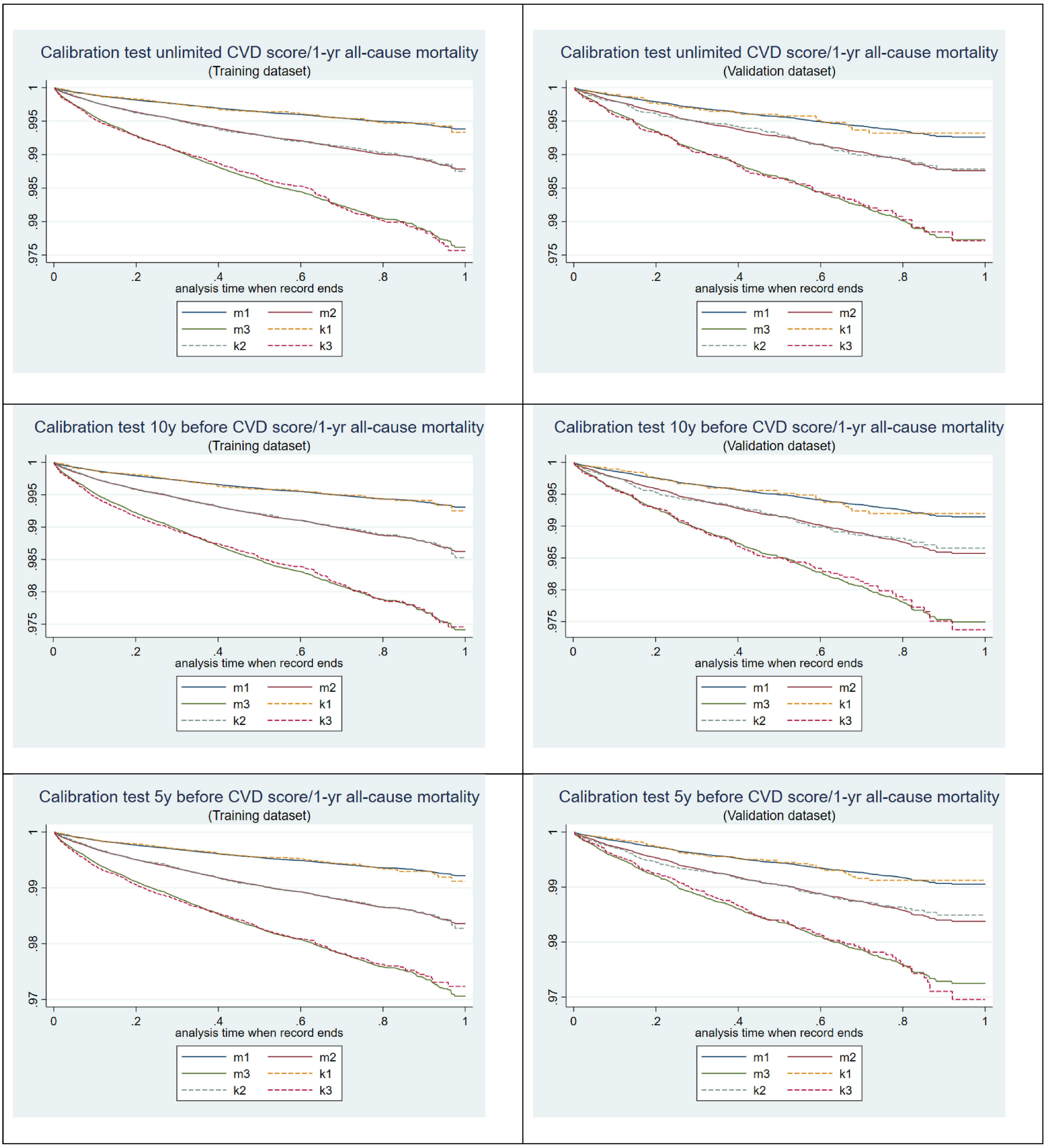

Supplementary data to '*Assessing the severity of CVD in 213,088 patients with CHD*'- *Zghebi et al. 2020*

Figure S4 Schoenfeld residuals for testing proportional hazards of fitted survival models using unlimited CV severity score (by gender) for 1-yr all-cause mortality – training dataset

a) using unlimited CV severity score

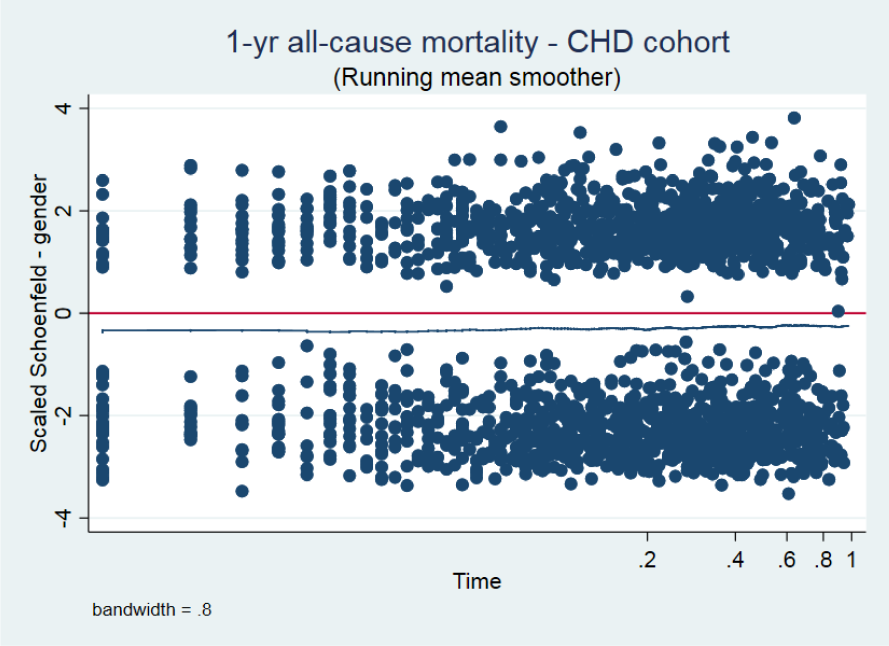

b) using socio-demographics variables only

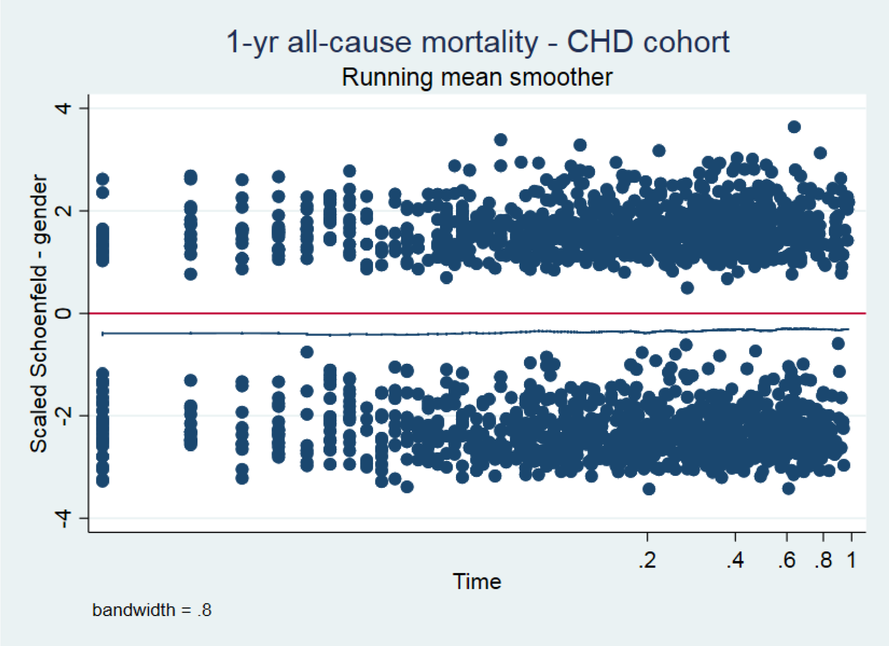

Supplementary data to '*Assessing the severity of CVD in 213,088 patients with CHD'- Zghebi et al. 2020*

Figure S5 Schoenfeld residuals for testing proportional hazards of fitted survival models using unlimited CV severity score (by gender) for1-yr 1-year CV/diabetes-related mortality – training dataset

a) using unlimited CV severity score

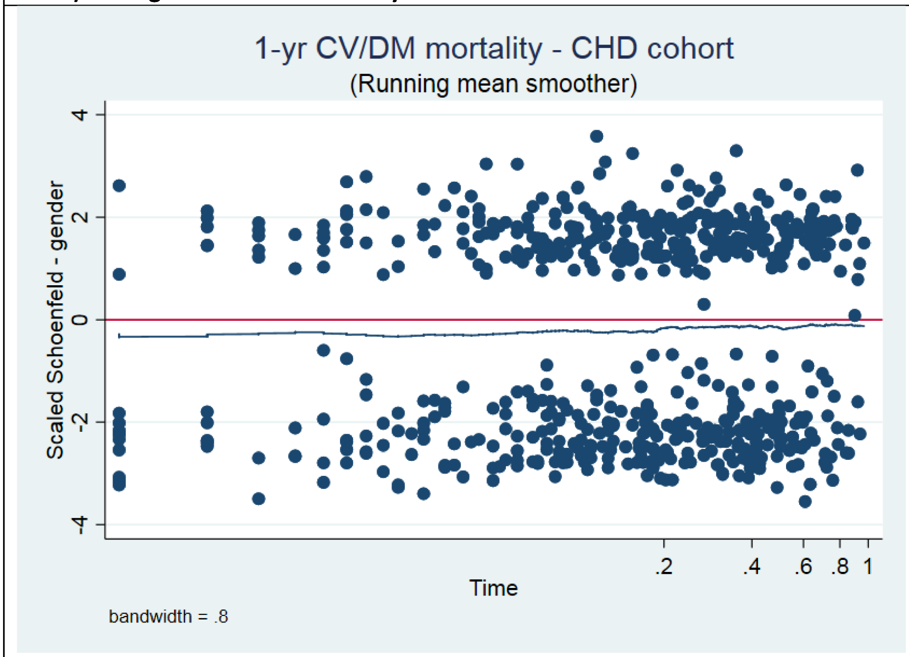

b) using socio-demographics variables only

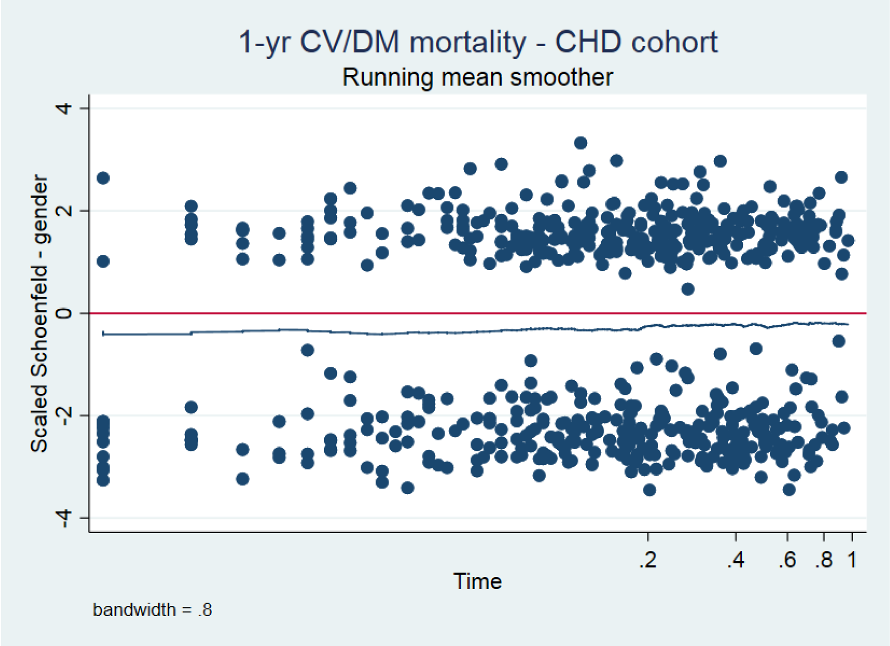

Supplementary data to '*Assessing the severity of CVD in 213,088 patients with CHD'- Zghebi et al. 2020*

Figure S6 Schoenfeld residuals for testing proportional hazards of fitted survival models using unlimited CV severity score (by gender) for 1-yr any cause hospitalisation – training dataset

a) using unlimited CV severity score

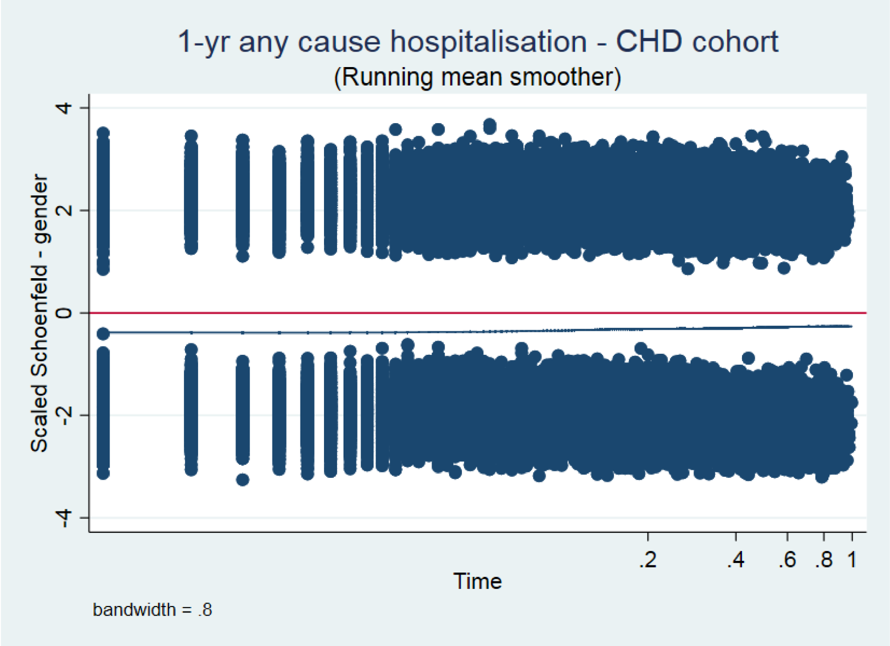

b) using socio-demographics variables only

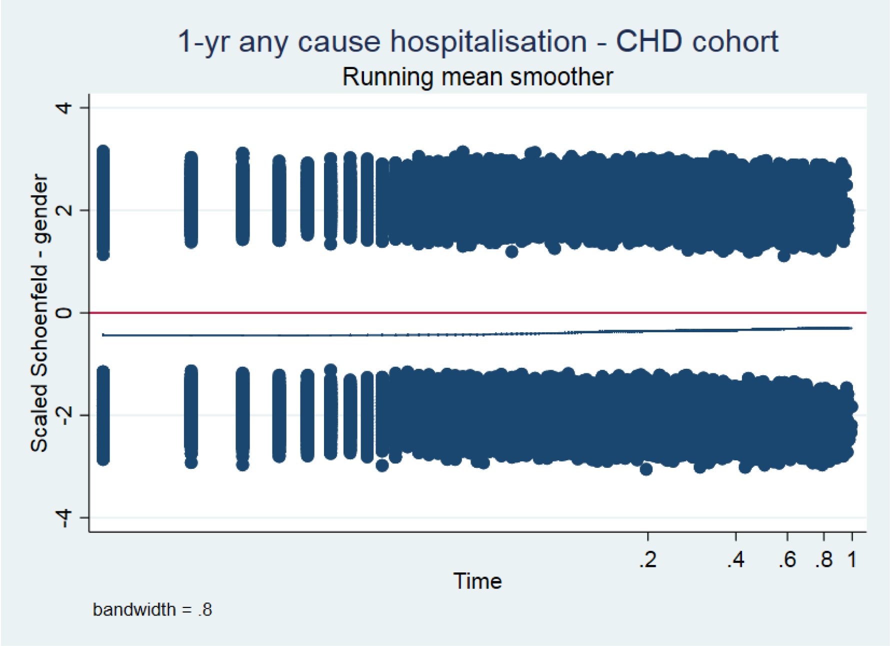

Supplementary data to '*Assessing the severity of CVD in 213,088 patients with CHD'- Zghebi et al. 2020*

Figure S7 Schoenfeld residuals for testing proportional hazards of fitted survival models using unlimited CV severity score (by gender) for 1-yr CV/diabetes-related hospitalisation – training dataset

a) using unlimited CV severity score

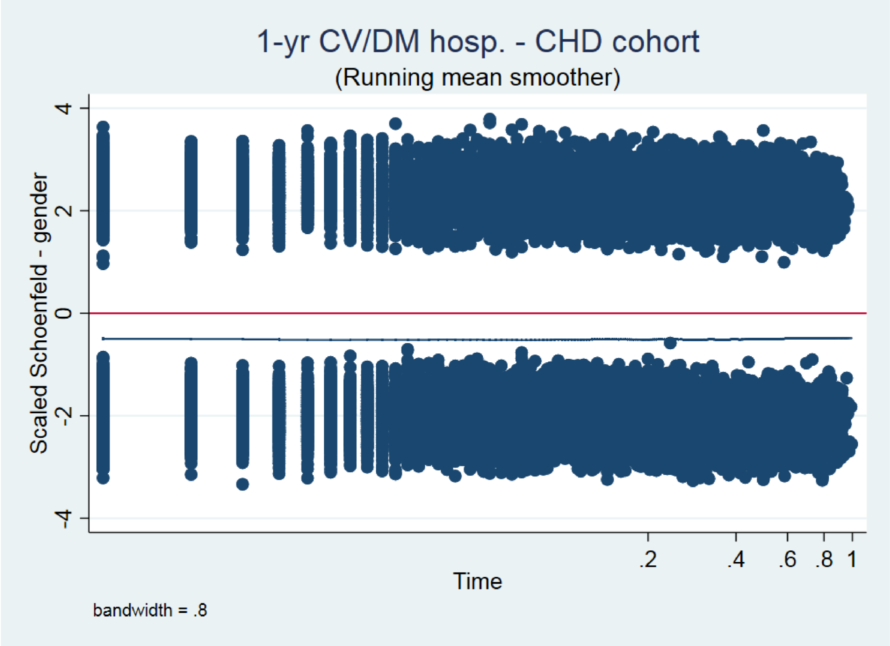

b) using socio-demographics variables only

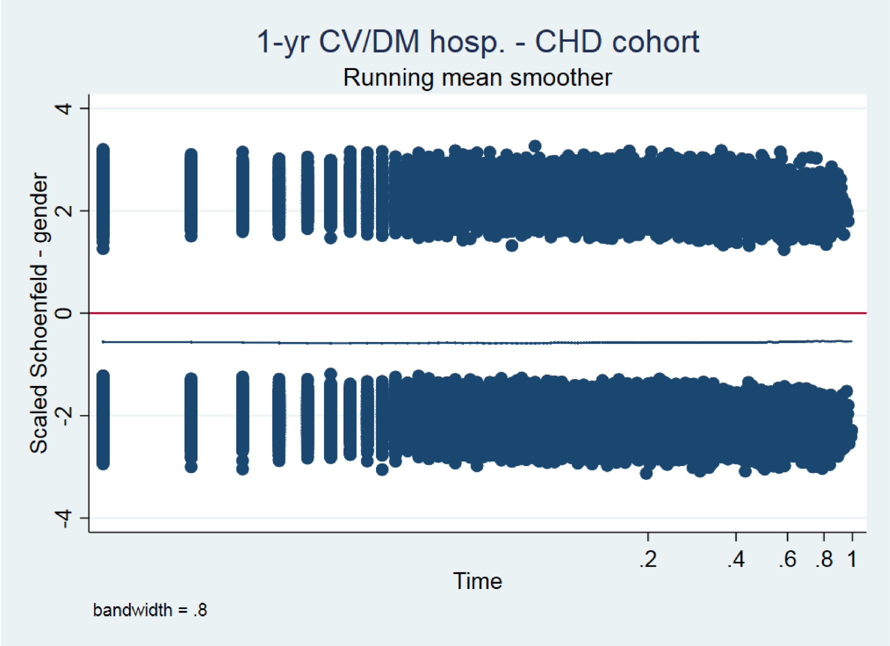

Supplementary data to '*Assessing the severity of CVD in 213,088 patients with CHD*'- *Zghebi et al. 2020*

Figure S8 Schoenfeld residuals for testing proportional hazards of fitted survival models using unlimited CV severity score (by gender) for 1-yr aggregated any hospitalisation or death – training dataset

a) using unlimited CV severity score

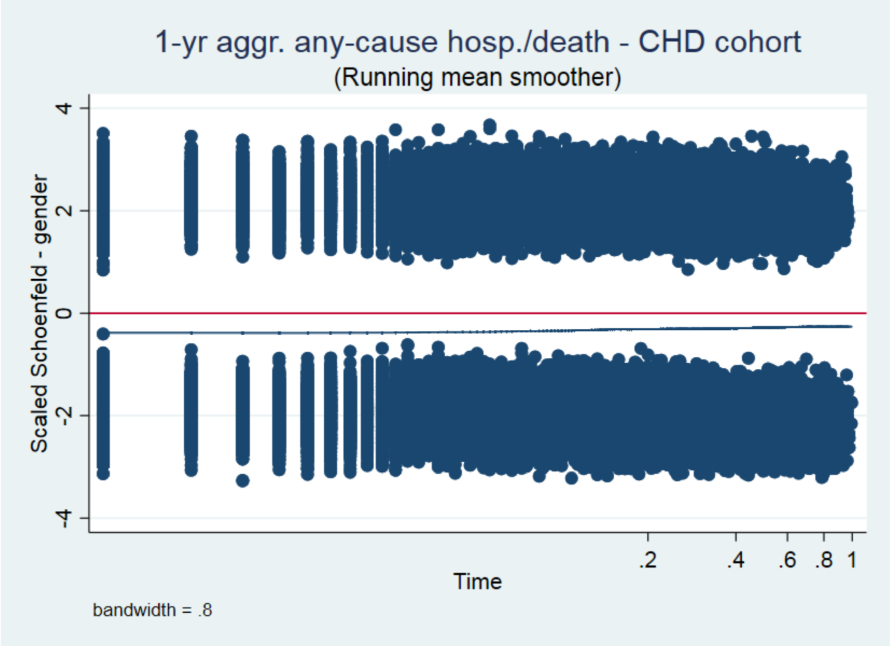

b) using socio-demographics variables only

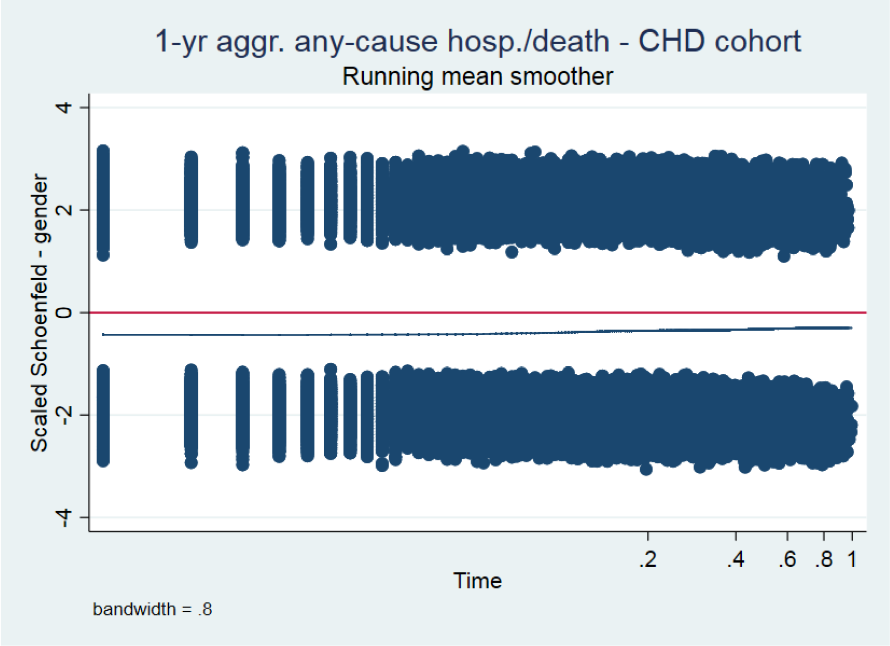

Supplementary data to '*Assessing the severity of CVD in 213,088 patients with CHD'- Zghebi et al. 2020*

Figure S9 Schoenfeld residuals for testing proportional hazards of fitted survival models using unlimited CV severity score (by gender) for 1-yr all-cause mortality – validation dataset

a) using unlimited CV severity score

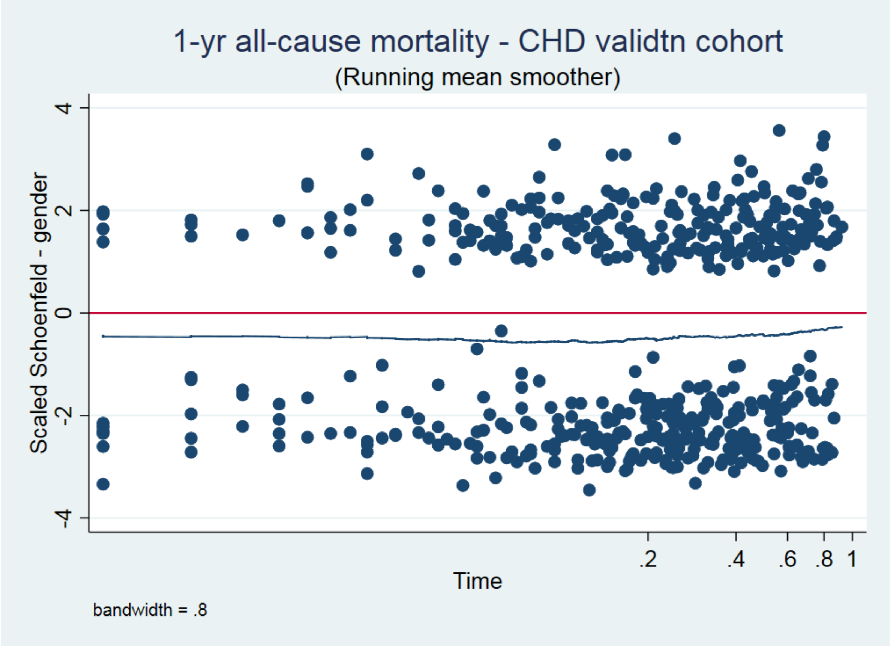

b) using socio-demographics variables only

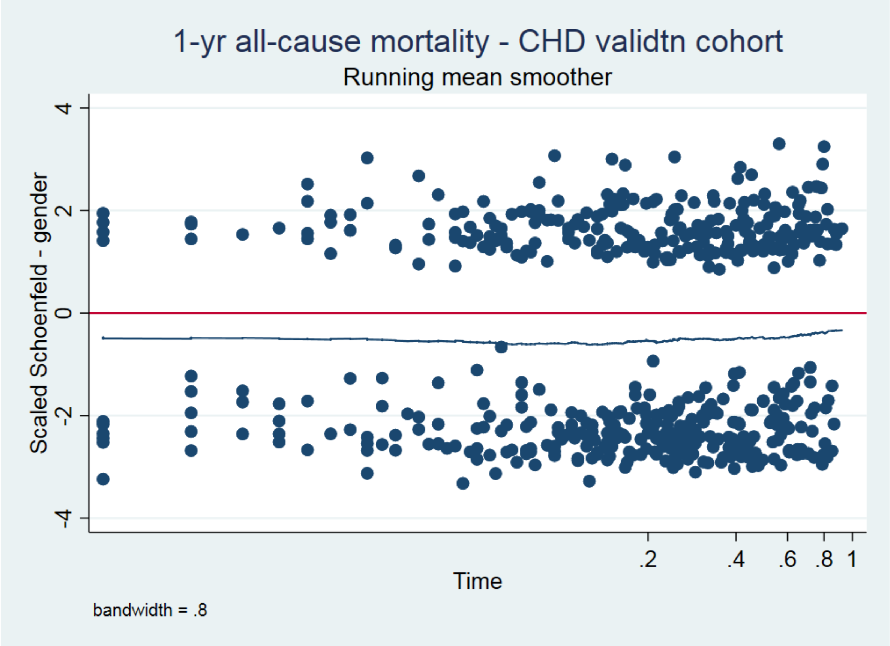

Supplementary data to '*Assessing the severity of CVD in 213,088 patients with CHD'- Zghebi et al. 2020*

Figure S10 Schoenfeld residuals for testing proportional hazards of fitted survival models using unlimited CV severity score (by gender) for1-yr 1-year CV/diabetes-related mortality – validation dataset

a) using unlimited CV severity score

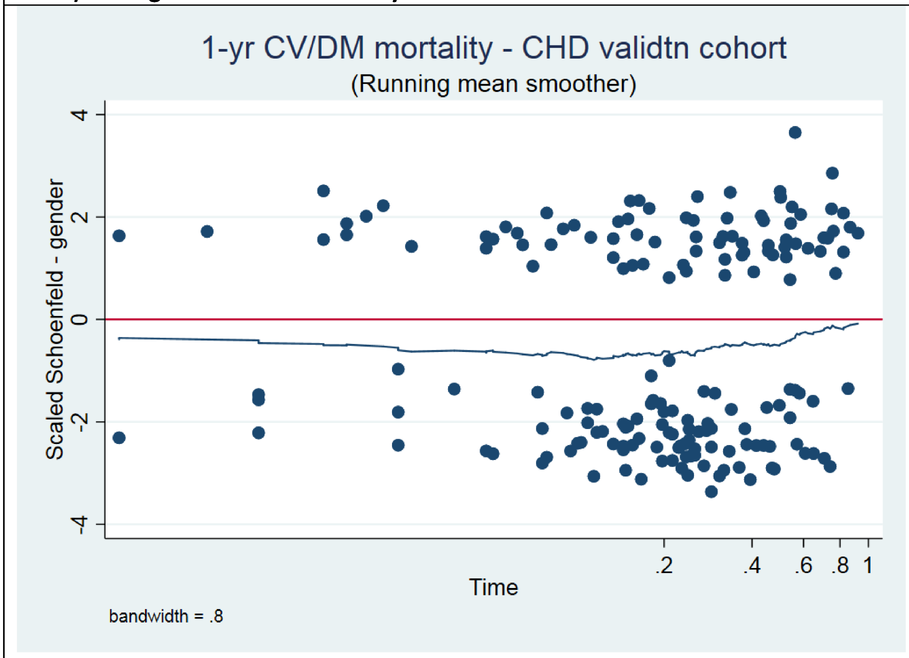

b) using socio-demographics variables only

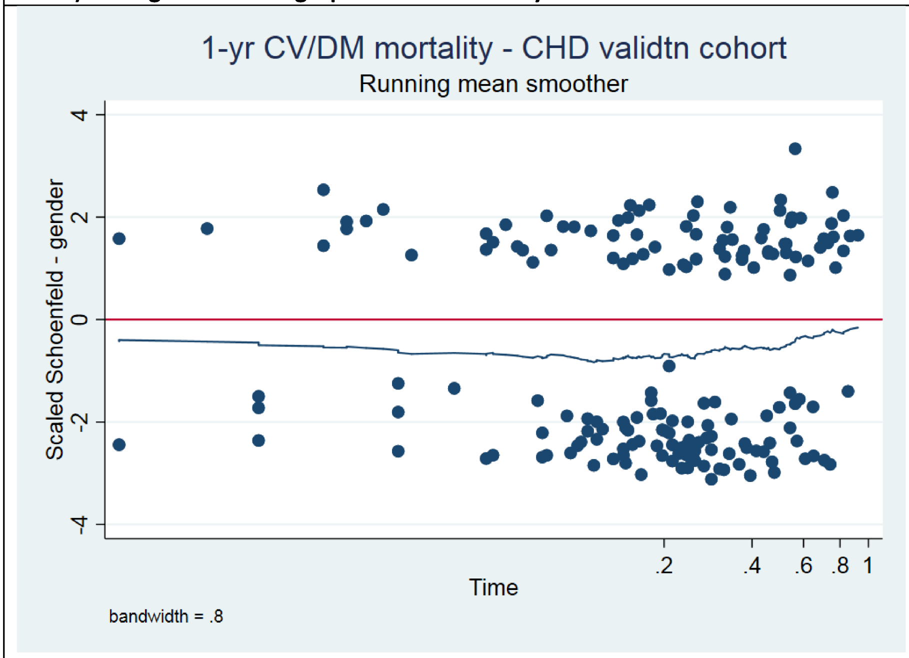

Supplementary data to *'Assessing the severity of CVD in 213,088 patients with CHD'- Zghebi et al. 2020*

Figure S11 Schoenfeld residuals for testing proportional hazards of fitted survival models using unlimited CV severity score (by gender) for 1-yr any cause hospitalisation – validation dataset

a) using unlimited CV severity score

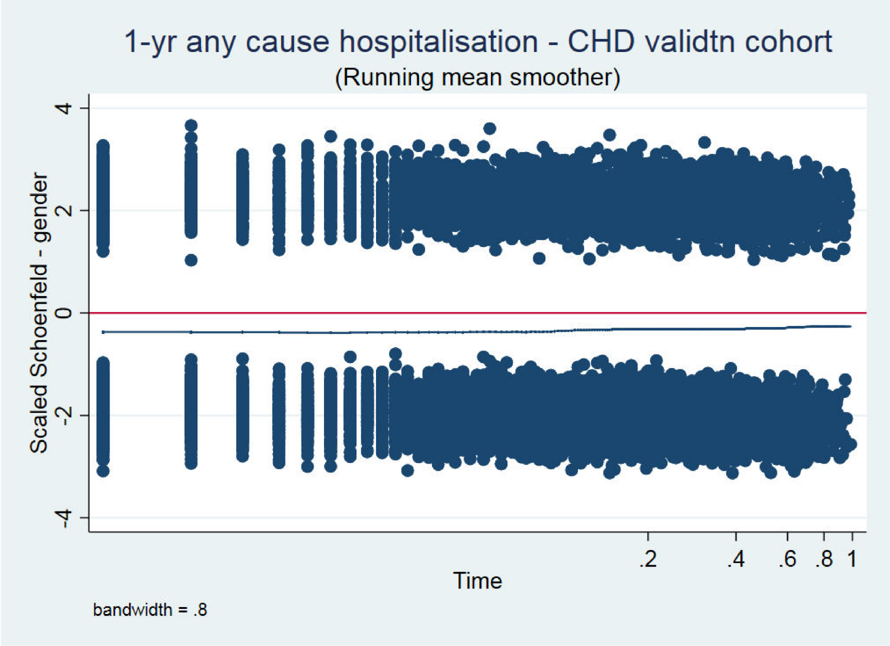

b) using socio-demographics variables only

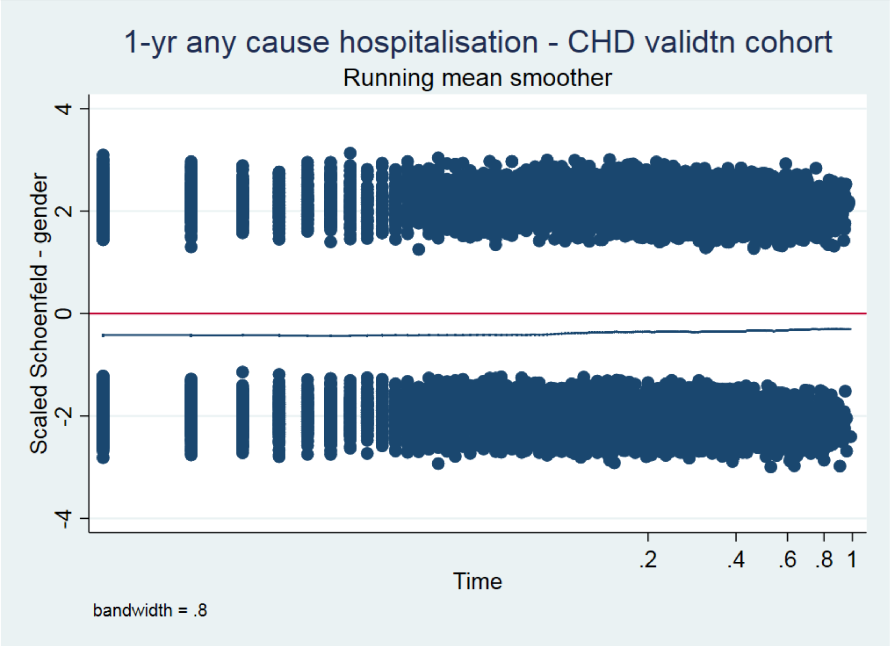

Supplementary data to '*Assessing the severity of CVD in 213,088 patients with CHD'- Zghebi et al. 2020*

Figure S12 Schoenfeld residuals for testing proportional hazards of fitted survival models using unlimited CV severity score (by gender) for 1-yr CV/diabetes-related hospitalisation – validation dataset

a) using unlimited CV severity score

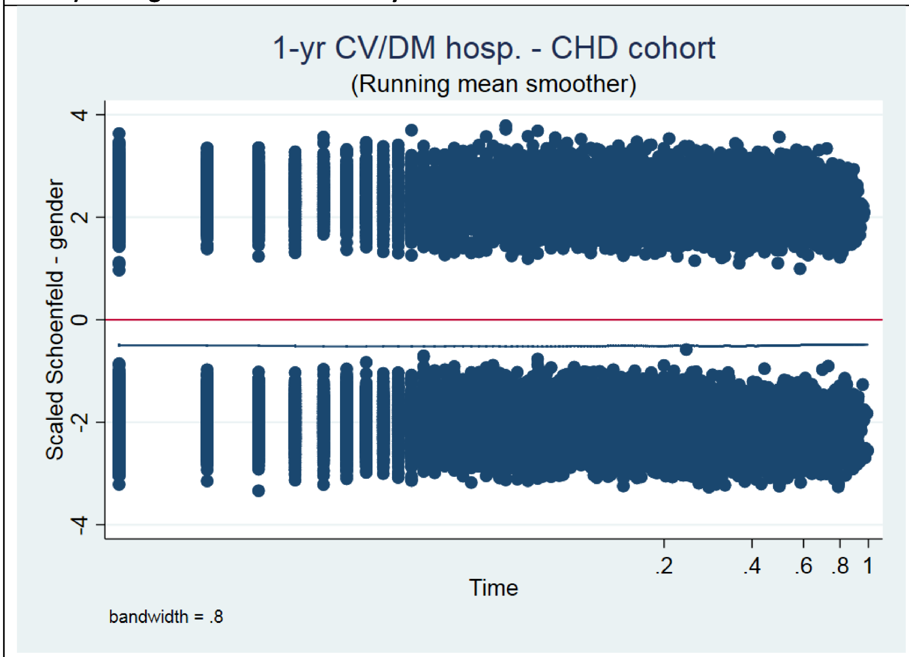

b) using socio-demographics variables only

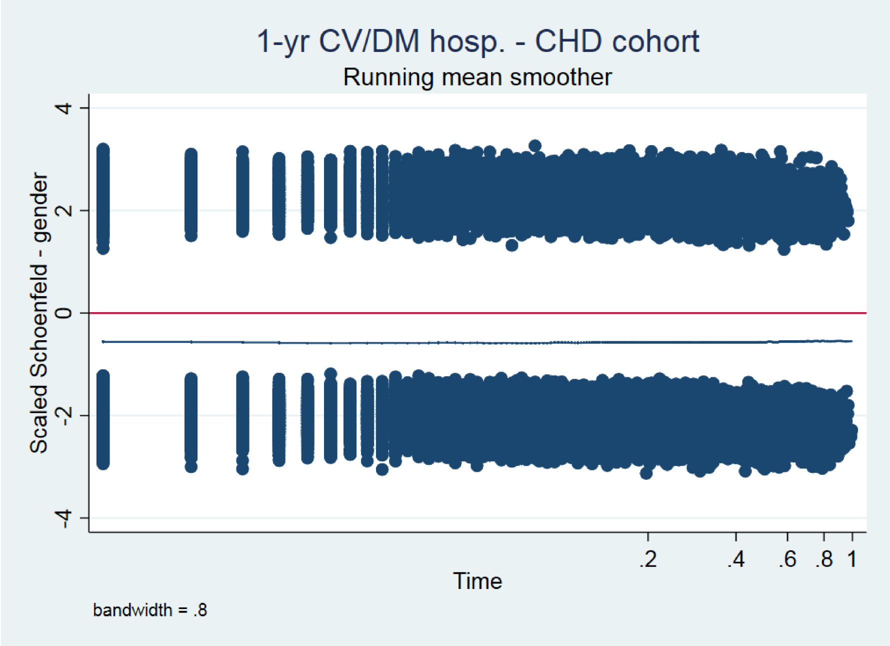

Supplementary data to '*Assessing the severity of CVD in 213,088 patients with CHD*'- *Zghebi et al. 2020*

Figure S13 Summary figure illustrating the study methodology and main findings

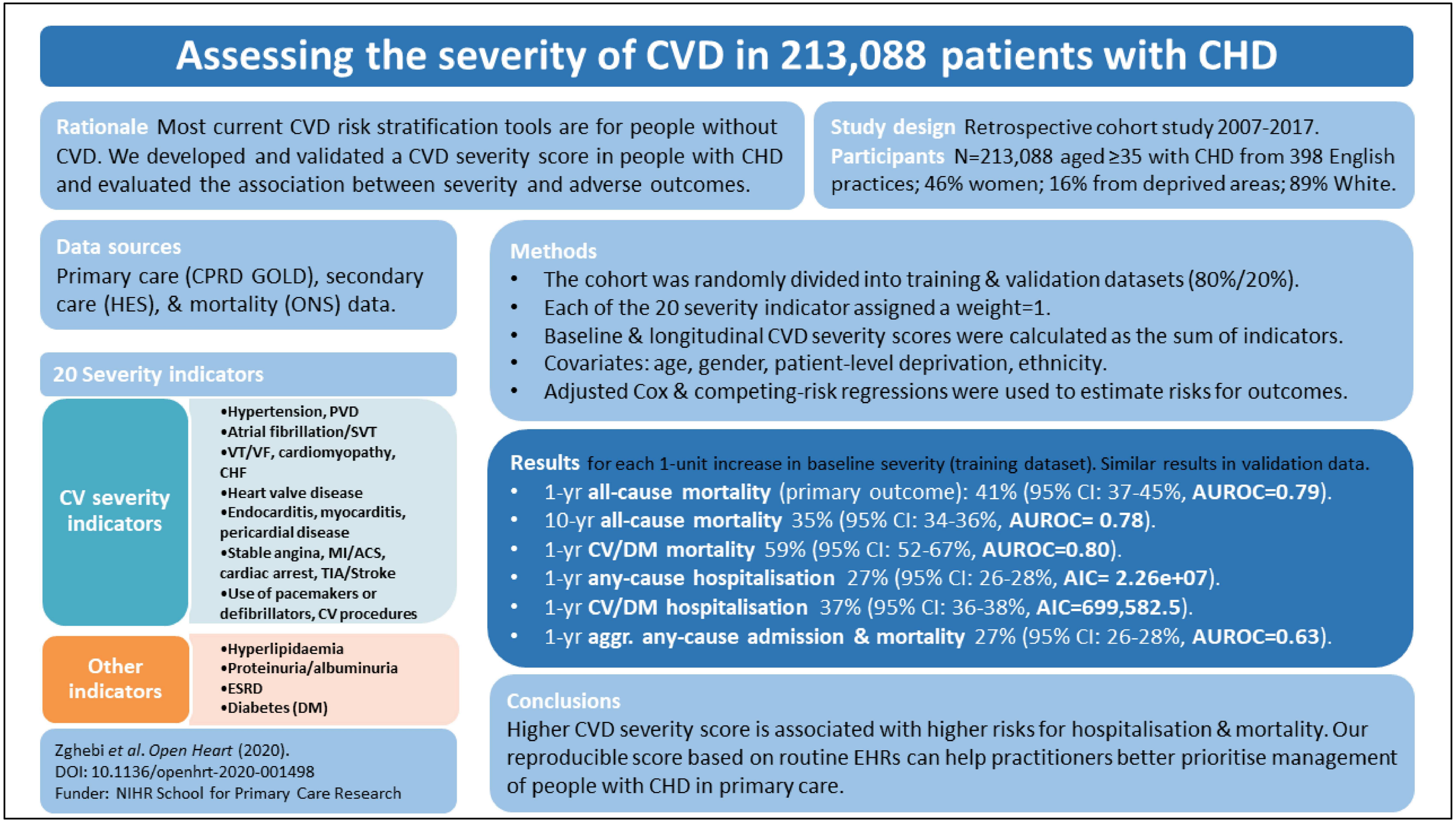

Supplementary data to '*Assessing the severity of CVD in 213,088 patients with CHD'- Zghebi et al. 2020*

Figure S14 Visual abstract of the study

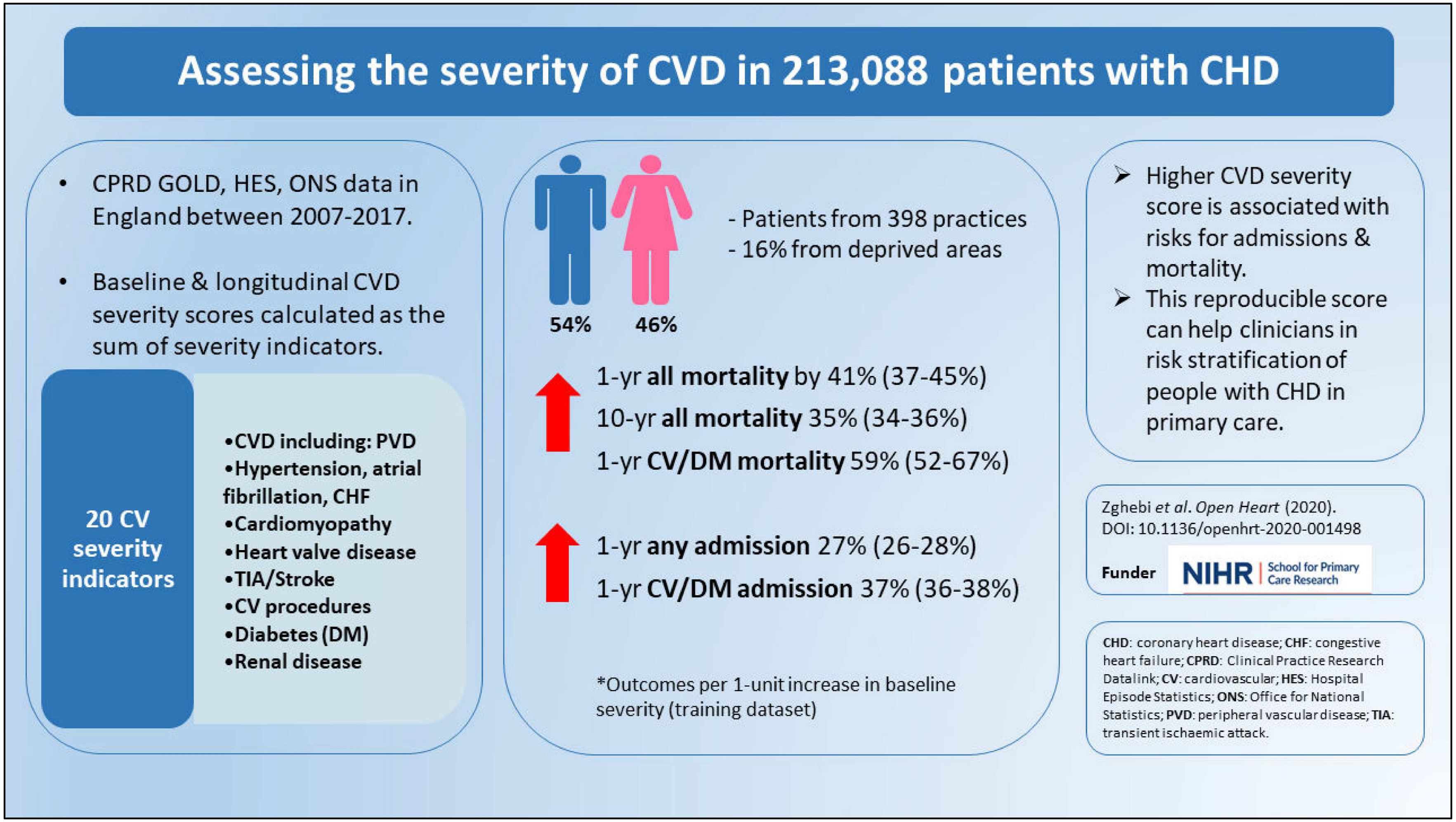

Supplement: Supplementary data [file openhrt-2020-001498supp001.pdf]
